# The effectiveness, feasibility, and acceptability of an education intervention promoting healthy lifestyle to reduce risk factors for metabolic syndrome, among office workers in Ethiopia: A protocol for a randomized control trial study

**DOI:** 10.1371/journal.pone.0307659

**Published:** 2024-08-30

**Authors:** Sitotaw Kerie Bogale, Haribondhu Sarma, Darren Gray, Matthew Kelly

**Affiliations:** 1 The National Centre for Epidemiology & Population Health, The Australian National University, Canberra, Australian Capital Territory, Australia; 2 College of Medicine and Health Sciences, Bahir Dar University, Bahir Dar, Ethiopia; 3 Population Health Program, QIMR Berghofer Medical Research Institute, Brisbane, Australia; University of Montenegro-Faculty of Medicine, MONTENEGRO

## Abstract

**Background:**

Nowadays, metabolic syndrome has become a major health threat, and affects over one billion people globally. It also plays a great role in the growth of diseases like type 2 diabetes, coronary diseases, stroke, and other chronicity. It increases the risk of cardiovascular disorder and stroke by three to ten times and diabetic mellitus by ten times. The prevalence of metabolic syndrome is increasing globally as a result of epidemiological shift. Low and middle-income countries are facing an increasing burden of metabolic syndrome. There is a need for concerted efforts to modify behavioral risk factors that significantly contribute to the prevalence of the syndrome. This can be done by developing and implementing appropriate interventions that can bring behavior change after testing for effectiveness, feasibility, and acceptability. Thus, this study aims to develop and test the effectiveness, feasibility and acceptability of an education intervention promoting healthy lifestyle to reduce risk factors for metabolic syndrome, among office workers in Ethiopia.

**Methods and analysis:**

This randomized controlled trial will be implemented with 226 bank employees (age ≥18 years) with metabolic syndrome from government and private banks in Bahir Dar City, Ethiopia. Participants will be randomized to intervention (education) and control (general health advice) groups. The intervention group will be given one-on -one base education about healthy diets, physical exercise, stress management, avoidance of harmful alcohol consumption and smoking cessation by experts on health promotion. Text messages will be sent every two weeks and reading materials will also be provided. Additionally, a review meeting will be held at the 3^rd^ and 6^th^ month of the intervention. The primary outcomes of interest will be change in metabolic parameters (obesity levels, blood pressure, fasting blood glucose, total cholesterol, high density lipoprotein, low density lipoprotein, and triglycerides). Secondary outcomes will be knowledge, attitudes and practice of the participants towards lifestyle and cardiovascular risk factors, feasibility, acceptability, implementation fidelity, and cost-effectiveness of the intervention. Data will be collected at three time points: at baseline, at the 6^th^ month of the intervention and at the end of the intervention (9 months). Generalized linear mixed models will be utilized to compare the desired outcome between the trial arms, after accounting for baseline variations. Cost-benefit analysis and a qualitative process evaluation of the intervention will also be conducted.

**Discussion:**

This randomized control trial study will provide information on the effectiveness, feasibility, and acceptability of an education intervention promoting healthy lifestyle to reduce risk factors for metabolic syndrome, among office workers in Ethiopia, where the burden of metabolic syndrome is high among office workers.

**Clinical trial registration:**

This trial has been prospectively registered at the Australian New Zealand Clinical Trials Registry: ACTRN12623000409673p.

## Introduction

Metabolic syndrome (MetS) is a collection of disorders that comprise: obesity, insulin resistance, glucose intolerance, high blood pressure and impaired regulation of body fat [1].

World Health Organization, National Cholesterol Education Program Adult Treatment Panel (NCEP: ATP III) and International Diabetic Federation (IDF) define the condition according to the guidelines in Table 1 [2].

**Table 1:** Criteria for metabolic syndrome definitions in adults.

World Health Organization (WHO)	National cholesterol education program adult treatment panel (NCEP: ATP III)	International-diabetic federation (IDF)
**Insulin resistance plus two of the following:** Abdominal obesity (waist-to-hip ratio > 0.9 in men or > 0.85 in women, or body mass index (BMI) > 30 kg/m2. Triglycerides 150 mg/dl or greater, and/or high-density lipoprotein (HDL)-cholesterol < 40 mg/dl in men and < 50 mg/dl in women. Blood pressure (BP) 140/90 mmHg or greater. Microalbuminuria (urinary albumin secretion rate 20 μg/min or greater, or albumin-to-creatinine ratio 30 mg/g or greater).	**Any three or more of the following:** Waist circumference > 102 cm in men, > 88 cm in women. Triglycerides 150 mg/dl or greater. HDL-cholesterol < 40 mg/dl in men and < 50 mg/dl in women. BP 130/85 mmHg or greater. Fasting glucose 110 mg/dl or greater.	**Central obesity plus two of the following:** Triglycerides 150 mg/dl or greater. HDL-cholesterol < 40 mg/dl in men and < 50 mg/dl in women. BP 130/85 mmHg or greater. Fasting glucose 100 mg/dl or greater.

Other health care organizations each have also their own definitions that only slightly differ from the definitions of the above health care organizations [2].

Metabolic syndrome has become a major health threat in the modern world. More than one billion people in the globe are now affected by metabolic syndrome and the condition plays an important role in the blowout of the diseases like type 2 diabetes, coronary diseases, stroke, and other chronicity [3]. It increases the risk of cardiovascular disorder and stroke by three to ten times and type 2 diabetes mellitus by ten times [4]. It also raises the expense of medical care [5]. People with metabolic risk factors incur healthcare expenses that are up to 20% more than those of patients without risk factors. They use outpatient and physician services more frequently, spend more money on drugs, and require longer hospital stays [6].

Metabolic syndrome is speedily expanding in the developing world [7, 8]. Sub Saharan Africa and the Middle East / North Africa are predicted to have the highest rates of diabetes increase over the next 25 years, at 141% and 104%, respectively [3]. This indicates that, the prevalence of metabolic syndrome will also increase by more than three times in the next 25 years in the continent.

The reasons for the increase of metabolic syndrome prevalence in Africa are thought to be due to a shift from traditional African to western lifestyles or the rise in the use of risk behaviors, such as consumption of unhealthy diet, reduced physical activity, harmful alcohol consumption and tobacco use, as well as due to the increment of urbanizations and increasing life expectancies [9, 10]. Globalization has also contributed a significant impact on the incidence of metabolic syndrome in sub-Saharan Africa via contributing to the availability of unhealthy diet, the expansion of urbanization, racial and cultural tensions, political upheaval and stress over time [11]. The development of metabolic syndrome is also greatly influenced by knowledge, attitudes, and practices (KAP) towards lifestyle and cardiovascular risk factors [12–14].

The status of metabolic syndrome in Ethiopia is similar to that in Africa more broadly. It is an emerging public health issue for the nation. According to a systematic review and meta-analysis study conducted in 2020, the pooled prevalence of metabolic syndrome in Ethiopian population is 34.89% and 27.92% according to ATPIII and IDF diagnostic criterions respectively [15]. In addition to consumption of high calorie-low fiber fast food and the decrease in physical activity, one of the main factors for the increment of metabolic syndrome is prolonged sitting and stress due to work behavior in Ethiopia [16–19]. Different studies confirmed that the prevalence of metabolic syndrome among office workers is high. A cross-sectional study conducted in Ethiopia in 2019 among 1,164 adults working in office revealed that, the prevalence of metabolic syndrome was 20.1% [18]. A study conducted in Ethiopia among 1,935 bank employees and teachers also confirmed that, the overall prevalence of metabolic syndrome was 12.5% and 17.9%, according to ATPIII and IDF respectively [20]. A cross sectional study conducted on the prevalence and associated factors of undiagnosed hypertension among bank workers in Bahir Dar city, Ethiopia, also showed that, the overall prevalence of undiagnosed hypertension among 513 bank workers was 24.8% [21]. Another cross-sectional study carried out among 368 bank employees in Bahir Dar, Gondar, and Dessie, Ethiopia, also found that the prevalence of hypertension was 52.4% [19]. That means bank workers are at greater risk for metabolic syndrome due stress and prolonged sitting at work, in addition to using mechanized transportations and consumption of high Calorie-low fiber fast food.

Given this increasing burden of metabolic syndrome in Ethiopia, there is an urgent need to develop effective and appropriate interventions that can bring behavior change after testing for effectiveness, feasibility, and acceptability [22, 23]. Education interventions for healthy lifestyle have been successful in many settings [24, 25]. However, most interventions for metabolic syndrome have only been tested in middle and high -income settings. According to a prospective, longitudinal, nonequivalent pretest-posttest comparison group design study conducted in San Jose, Costa Rica and Chiapas, Mexico to evaluate the impact of education intervention for healthy lifestyle to reduce cardiovascular diseases confirmed that there was a significant improvement of behavior change in the intervention group [25]. But we do not know this will be equally effective or not in Ethiopia, and it is essential to test the specific effectiveness, feasibility and acceptability in Ethiopia. As well, the level of knowledge, attitudes and practice towards lifestyle and cardiovascular risk factors among bank workers with metabolic syndrome in Ethiopia, have not been investigated.

Thus, this study will provide evidence about the effectiveness, feasibility and acceptability of lifestyle education intervention on the metabolic syndrome, the level of knowledge, attitudes and practice towards lifestyle and cardiovascular risk factors among bank employees with metabolic syndrome in Ethiopia. Consequently, the evidence will be used for policy makers to scale up this management strategy and for stakeholders, as an input to plan effective metabolic syndrome management strategy, and will also be used as a reference for other randomized control trial study. Moreover, it will be used as evidence for professionals to apply this intervention for risky groups.

## Objectives

➢ To develop and test the effectiveness of a lifestyle education intervention on metabolic syndrome management among bank employees in Ethiopia.
➢ To assess and compare the levels of knowledge, attitudes, and practices towards lifestyle and cardiovascular risk factors before and after the intervention as well as between the control and intervention groups among bank employees with metabolic syndrome in Ethiopia.
➢ To assess the feasibility, and acceptability of an education intervention for healthy lifestyle on metabolic syndrome management among bank employees in Ethiopia.
➢ To evaluate the cost-benefit of the educational intervention promoting healthy lifestyle to reduce risk factors for metabolic syndrome among bank employees in Ethiopia.
➢ To evaluate the process of education intervention promoting healthy lifestyle to reduce risk factors for metabolic syndrome for bank employees in Ethiopia.

## Methods

### Study design

A randomized control trial using a parallel design will be employed to develop and test the effectiveness, feasibility, and acceptability of lifestyle intervention on metabolic syndrome management, and to assess and compare the levels of knowledge, attitudes, and practices towards lifestyle and cardiovascular risk factors before and after the intervention as well as between the control and intervention groups among bank employees with metabolic syndrome, and also to evaluate the cost effectiveness and the implementation process of the intervention. The trial has been designed according to the Standard Protocol Items: Recommendations for Interventional Trials (SPIRIT) (**S1 File**). It is registered with the Australian New Zealand Clinical Trials Registry (ACTRN12623000409673p).

### Study setting

The study will be conducted in the Bahir Dar city, Ethiopia, among bank workers. Bahir Dar is located 565 km Northwest of Addis Ababa and it is the capital city of the Amhara regional state. There are 210 bank branches with 2,310 workers in the city.

### Participants and recruitment

The participants of this trial will be employees of both private and government banks in Bahir Dar City, Ethiopia. Bank employees are chosen as participants in this intervention trial because studies done among bank employees in Ethiopia revealed that the prevalence of metabolic syndrome is significant and that rapid intervention is needed for this population [19–21]. For instance, a cross-sectional study conducted in Ethiopia among 1,935 bank employees and teachers found that 12.5% of participants had metabolic syndrome, and it recommended for more efforts to screen for, identify, and treat MetS and its components among Ethiopian bank employees [20]. A cross-sectional study conducted on the prevalence and contributing factors of undiagnosed hypertension among bank employees in Bahir Dar city, Ethiopia, also found that the prevalence of undiagnosed hypertension among 513 bank employees was 24.8%, and that low levels of knowledge about hypertension and physical inactivity were the main risk factors for the development of hypertension. This study suggested that creating awareness, frequent screening and apply the right interventions for this vulnerable group is crucial [21]. Another cross-sectional study done among 368 bank employees in Amhara metropolitan cities (Bahir Dar, Gondar, and Dessie) also found that the prevalence of hypertension was 52.4%, and that variables such as overweight and obesity, daily fruit consumption, moderate to vigorous physical activity, the presence of stressful events, and inadequate cardiovascular diseases knowledge were all linked to hypertension [19]. This study recommended that raising awareness about cardiovascular disorders and behavior change interventions that enhance bank workers’ engagement in physical exercise, and a healthy diet is urgently required for this group of population. The other reasons to select bank workers as participants is the fact that bank employees have a generally stable workforce and are willing to participating in the study are the other reason used to choose participants.

The following methods will be applied to contact and recruit participants: The district headquarters of each bank will get an official letter from the health department of the city administration, after which letters will be issued to each bank branch from the head office of the bank and communications will be made between the managers of each branch and the bank employees. Then, participants will be approached and recruited after being informed about the objectives of the study, inclusion and exclusion criteria, the potential benefits and risks of participation, and ethical considerations of the study. Finally, prior to the start of data collection, written informed consent will be obtained from each participant. Then, the participant will be recruited and screened based on their level of obesity and blood pressure, and those who meet at least one of the two criteria (obesity or high blood pressure) will be included in the trial and will have test for biomarkers (Fasting blood glucose level, Total cholesterol, High density lipoprotein cholesterol, low density lipoprotein cholesterol, and Triglyceride).

To make recruiting effective and to increase participant willingness, the objectives of the study, inclusion and exclusion criteria, ethical considerations of the study, the advantages of the study and their rights even to stop in the middle of the procedure will be explained to participants. The participants will be informed that their participation in this study is very important for the success of the study and for paving the way for the development of the policy in this area. Additionally, they will be told that there is no risk except some discomforts to them in taking part in this study, and that all data obtained from them would be kept confidential via a password with no requirement that their identities be recorded, and results will also be communicated to them at the end of the study. They will also be told that even if they do not meet the requirements to participate in the study, they will still get advice on living a healthy lifestyle and about seeking medical counsel if they exhibit any signs of metabolic syndrome.

### Inclusion and exclusion criteria

#### Inclusion criteria

Bank workers with age ≥18 years old and who fulfil at least one of the following National Cholesterol Education Program Adult Treatment Panel III (NCEP: ATPIII) metabolic syndrome criteria; Waist circumference >102 cm in men, > 88 cm in women, Triglycerides ≥150 mg/dl, HDL-cholesterol < 40 mg/dl in men and < 50 mg/dl in women, BP ≥130/85 mmHg, fasting glucose ≥110 mg/dl will be included in the study.

#### Exclusion criteria

Bank workers who have at least one of the NCEP: ATP III metabolic syndrome criteria but with dietary restrictions and absolute contraindication for physical activity due to musculoskeletal, neurological, vascular, lung and cardiac problems, pregnant mothers, those who have a plan to be pregnant within the intervention months and diagnosis of severe psychiatric disorders, significant cognitive impairment, and those who will not available throughout the program will be excluded from the study.

### Sample size

Because researchers can use mean and standard deviation (SD) from previous studies in a similar context to estimate sample size [26]. We utilized the mean and SD from a study at Jimma University among university staff to evaluate the effectiveness of behavior change communication on metabolic syndrome among Ethiopian adults in a randomized controlled trial [27]. The sample size for this randomized control trial study was determined under the assumptions and using the formula of the sample size calculation for randomized control trial studies with continuous outcome variables [26]. Since blood pressure is the study’s primary outcome measure and the most common indicator of metabolic syndrome among bank employees in Bahir Dar city and also gives a maximum sample size among other indicators, the means and standard deviations of systolic blood pressure were taken from a study with a similar design that was conducted in Ethiopia [27], and a power of 80%, a 1:1 ratio, and a 95% confidence interval were used. Then the sample size was calculated as follows;

$$n1=\frac{(\sigma 1^{2}+\sigma 2^{2}){\;[Z1‐\alpha /2+Z\;1‐\beta ]}^{2}}{{\left(M1‐M2\right)}^{2}}$$

$$n1=\frac{(15.0^{2}+16.3^{2})\;{\left[1.96+0.84\right]}^{2}}{{\left(107.8‐114.2\right)}^{2}}=\frac{(225+265.69)\;{\left[2.8\right]}^{2}}{6.4^{2}}=\frac{\left(490.69)(7.84\right)}{40.96}=94$$

n1 = 94 and n2 = n1*1 = 94, Therefore, N = n1+n2 = 188

where,

m1 = mean of systolic blood pressure for the intervention group

m2 = mean of systolic blood pressure for the control group

σ1 = standard deviation of systolic blood pressure for the intervention group

σ2 = standard deviation of systolic blood pressure for the control group

n1 = sample size for group 1

n2 = sample size for group 2

α = probability of type I error (usually 0.05)

β = probability of type II error (usually 0.2)

z = critical Z value for a given α or β

N = Total sample size

By taking into account a 20% attrition rate, 226 total individuals or 113 individuals per group will be recruited for the study.

### Randomization

Bahir Dar city is divided in to two parts by the Nile River. From these two parts of the city, one will be randomly selected as the control area using a coin tossing randomization procedure by a principal investigator, and the other will assign as the intervention site. To reduce information contamination, bank employees who work in the control area will make up the control group, whereas bank employees who work in the intervention area will make up the intervention group. The data collectors, the nurse who will offer general advice to the control group, the education provider for intervention group, and the participants will all be blinded to the group assignment. Finally, each individual participant will be screened based on the inclusion and exclusion criteria, up until the target sample size is reached.

### Intervention: Education about healthy lifestyle

The intervention will comprise education about healthy lifestyle that includes the concept of metabolic syndrome, risk factors and its prevention, the importance of lifestyle modification such as healthy diet, physical exercise, avoidance of harmful alcohol consumption, smoking cessation and stress management, as well as motivation for changing behavior. The education intervention will be delivered by experts on health education and promotion after the education package is assessed and validated by a nutritionist, physical therapist and psychologist. The education will be delivered in a one-on-one basis at their office or at any other suitable places. Every two weeks, the researcher will send a text message to the participants as a reminder asking whether they have followed the suggested healthy diet, physical activity, and mechanisms of stress management, avoidance of harmful alcohol consumption and smoking. It will also enquire as to whether they recorded their actions using the provided self-report template **(S2 File).** Throughout the intervention, the participants will be encouraged to respond to the messages, ask questions, and seek guidance. Reading materials (Handout) will also be given to each participant with the key messages of “avoid the three whites including: fat, sugar and salt, have a healthy diet, aerobic exercise, avoid sources of trans fats, avoid smoking, avoid unsafe intake of alcohol (≥ 2 drinks per day), manage your stress and avoid sitting for long time”. Additionally, a review meeting with participants will be held at the 3^rd^ and 6^th^ month of the intervention to review the key points of the intervention and reinforce, enhancing health literacy, skills, and motivation among the participants, foster encouragement and support to enhance participants’ commitment and adherence to the intervention, and reassess and identify any obstacles encountered during the intervention, and collaboratively develop strategies to overcome these barriers to change. The primary investigator will supervise the education process. Adherence to the intervention will be improved and monitored by self-report checklists, attendance at review meetings, and text message answers.

### Development of interventions

#### Dietary intervention

The World Health Organization’s recommendation for adults will help as the basis for the development of the nutritional intervention. According to the recommendation, fruit, vegetable, legumes like lentils and beans, nuts, and whole grains, such as unprocessed maize, millet, oats, wheat, and brown rice, a daily intake of at least 400g, or five portions of fruit and vegetables, excluding starchy roots like potatoes, sweet potatoes, and cassava are all parts of a healthy diet.

#### Physical intervention

The physical intervention will be designed based on the World Health Organization’s recommendation for adults aged 18–64 years. WHO recommend adults aged 18–64 years should do at least 150–300 minutes of moderate-intensity aerobic physical activity; or at least 75–150 minutes of vigorous-intensity aerobic physical activity; or an equivalent combination of moderate-and vigorous-intensity activity throughout the week.

#### Psychological intervention

Psychological intervention which includes how they manage stress, avoid the use of harmful alcohol consumption and quit smoking will be prepared based on the theory of planned behavior. Each intervention component will be assessed and validate by the experts of the field.

### Control group: General health advice

The comparator is general health advices that will be given in an individual form by the nursing professional on physical exercising, healthy diet, self-care and monitoring, according to the national guidelines recommended for non-pharmacological managements for each component of the metabolic syndrome. Education package and reading material (handout) will also be provided for them at the end of the study.

### Outcome measures

We will collect outcome measures in three waves: (at the base line, at 6^th^ and 9^th^ month of the intervention). The data collectors will be the trained BSc nurses and laboratory technologist.

#### At the baseline

Socio-demographic data, metabolic syndrome parameters and knowledge, attitude and practice (KAP) will be collected from both groups.

#### At 6^th^ month

Feasibility and acceptability of the intervention will be measured from interventional group only.

#### At 9^th^ month

Metabolic syndrome parameters and KAP will be measured from both groups.

### Independent variables

Age, sex, marital status, educational status, work experience, working hours per day, monthly income, religion, and health condition will be the independent variables of the study and will be measured by the questionnaire at the baseline.

### Dependent variables

#### Primary outcomes

*Body size (Central obesity)*. These will be defined as waist circumference > 102 cm in men, > 88 cm in women [2], and will be measured with a tape measure at the approximate midpoint between the lower margin of the last palpable rib and the top of the iliac crest or at the abdomen’s maximum extension. It will be measured at the baseline and 9^th^ month of the intervention.

*Blood pressure (BP)*. Which is defined as BP ≥ 130/85 mmHg [2, 28], and will be measured as follows: Three blood pressure measurements will be taken using appropriate size sphygmomanometer after the person is taking a rest for 5 minutes for the first measurement and with a difference of 5 minutes between successive measurements. The mean systolic and diastolic BP measurements will be considered for interpretation. The measurement will be taken at the baseline and the 9^th^ month of the intervention.

*Biochemical markers*. Fasting blood glucose (FG), high density lipoprotein (HDL), and triglycerides (TGL)) which are defined as fasting glucose ≥110 mg/dl, HDL-cholesterol < 40 mg/dl in men and < 50 mg/dl in women and Triglycerides ≥150 mg/dl respectively [2]. From each eligible individual 5 ml of venous blood will be collected at baseline and at the end of the intervention before breakfast by employing infection prevention procedures. Plasma and serum will be separated by laboratory technologist and stored at -80°C for later analyses at Bahir Dar University teaching hospital laboratory department. The tests will analyze FG, total cholesterol, HDL-C and TGL, while low-density lipoprotein will be determined indirectly.

#### Secondary outcomes

Knowledge, attitudes and practice of the participants, feasibility, acceptability, cost benefit and implementation process will be the secondary outcomes of the study.

Knowledge and attitudes towards lifestyle and cardiovascular risk factors will be assessed using a valid 43-item questionnaire which was developed and tested in India [12], and its Cronbach’s alpha for the “knowledge,” and “attitude,” are 0.72, 0.8, respectively. This questionnaire has been validated and utilized for evaluating knowledge and attitudes regarding lifestyle and cardiovascular risk factors in several studies in Ethiopia, where it was employed separately for assessing hypertension, diabetes mellitus, and lifestyle [29–32]. To simplify the citation process, we have opted to reference the tool that was developed and tested in India, as it encompasses a comprehensive questionnaire and is easily citable.

*Knowledge*. Will be assessed with 20 questions. The answers will either be recorded as a correct answer (1), an incorrect answer, or I don’t know (0). The maximum possible score is 20, and the knowledge levels will break down into Poor knowledge score (1–7), Average knowledge score (8–14), and good knowledge score (15–20) [12].

*Attitudes*. Will be assessed using 13 questions. The answers will be recorded on a 0–2 Likert scale as disagree (0), agree (1), and strongly agree (2). The highest score is 26, and the categories for attitude will: Poor attitude (scores of 0–9), Average attitude (scores of 10–18), and good attitude (scores of 19–26) [12].

*Dietary habit*, *alcohol consumption*, *smoking and physical exercise practice*. Will be evaluated using short form food frequency questionnaire. The cut-offs for fruit and vegetable servings will be as follows; Fruit: < = 2 servings/wk = 1, >2 servings /wk and < 2 servings/day = 2, > = 2 servings/day = 3, Vegetables: < = 1 servings/day = 1, 1–3 servings/day = 2, > = 3 servings/day = 3 [33].

*Stress*. Will be measured by using perceived stress scale. It is developed in 1983 and it has 10 items with total score of 40 [34].

- Scores ranging from 0–13 would be considered low stress.
- Scores ranging from 14–26 would be considered intermediate stress.
- Scores ranging from 27–40 would be considered high stress.

**Feasibility** is the extent to which a new treatment, or an innovation, can be successfully used or carried out within a given agency or setting [35]. For feasibility, the criterion is practical. An intervention can be judged feasible if a task or an action can be performed relatively easily or conveniently given existing resources and circumstances like effort, time, and money, timing or sociopolitical will. It will be measured by Feasibility of Intervention Measure (FIM) [30]. These are 4-item tool for each outcome variables on a 5-point Likert scale, ranges from “Completely Disagree-Completely Agree” and score is calculated mean.

**Acceptability** is the perception among implementation stakeholders that a given treatment, service, practice, or innovation is agreeable, palatable, or satisfactory [35].

For acceptability, the criterion is personal. Two individuals can have different judgments regarding the acceptability of the same intervention based on their distinct needs, preferences, or expectations. It will be measured by Acceptability of Intervention Measure (AIM) [36]. These are 4-item tool for each outcome variables on a 5-point Likert scale, ranges from “Completely Disagree-Completely Agree” and score is calculated mean. Additionally, Sekhon’s theoretical framework for acceptability, which takes into account affective attitude, burden, ethicality, intervention coherence, opportunity costs, perceived effectiveness, and self-efficacy, will be used to evaluate acceptability from the perspectives of both deliverers and recipients.

### Cost benefit evaluation

The cost benefit evaluation will be assessed by collecting the cost data for the intervention implementation activities, which will be split into two categories: start-up costs (i.e., for screening, material preparation for education) and implementation costs (i.e., conducting educational sessions, monitoring). The cost benefit analysis will be made and the benefit would be outcome variables of the RCT and at the end we will compare them to how much costs are required for per unit of benefit. The cost benefit evaluation can be conducted separately for each outcome indicator of interest.

### Process evaluation

Concurrent process evaluation design will be implemented to evaluate the process of education intervention for promoting healthy lifestyle to reduce risk factors for metabolic syndrome, among office workers in Ethiopia.

**Fidelity** is defined as the extent to which an intervention is delivered as proposed. It will be checked based on the five main dimensions (Adherence, exposure or dose, quality of delivery, participant responsiveness and program differentiation of the interventions [35, 37]. It will be evaluated by participants and providers self-reports and researcher observations using the prepared checklist **(S3 File)**.

**Implementation process** will be evaluated using the Consolidated Framework for Implementation Research model [38]. The model contains five domains, including intervention, outer setting, inner setting, individuals involved and process. These five dimensions will be used to evaluate the study’s implementation process. Participant self-report method will be used to collect the data. Self-report format will be provided to each participant and they will be requested to list down the barriers they faced while they implement the intervention instructions **(S2 File).** If the data that are collected by self-report are not sufficient, interview will be implemented to collect further data at the end of the trial until data saturation and the interview topic guide will be informed by the Consolidated Framework for Implementation Research (CFIR) [32]. Then barriers will be identified for future successful large-scale randomized control trials.

### Data collection and safety monitoring

The trial data safety and adverse effect monitoring committee (DSMC) will be established to monitor the adverse effects of the intervention on participants. Experts in health promotion, laboratory technologists, nurses, and officials will make up the committee. Because the intervention won’t directly involve any invasive procedures or administer drugs that could affect study participants, there won’t be any potential for adverse risks or harms. The DSMC will be used as the protocol for data reporting that minimize bias. The principal investigator will keep track of all adverse events and connect them to the appropriate local health organization if any.

Data will be collected by trained BSc nurses and Laboratory technologist. Waist circumference, blood pressure, and blood sample will be taken at the workplace or another location the participant chooses. Data on knowledge, attitude, and practice about a healthy lifestyle and cardiovascular risk factors will be collected at their office. As well data on the feasibility and acceptability of the intervention will be collected at their office during the review meeting that will be held at the 6^th^ month of the intervention.

The data will only be accessible to the research team, and identifiable information will be kept apart from the rest of the data. Unless the participants have chosen differently, results will only be reported in collective and will not identify them specifically. Data will be securely stored on password-protected computers in the National Centre for Epidemiology and Population Health at the Australian National University. Hard copies of records will be kept in a locked filing cabinet in my office. All research data will be kept for at least five years after publications resulting from the study and securely stored. Following the storage term, all personally identifying information will be eliminated from the data, which will then be archived at the Australian Data Archive (www.ada.edu.au) for use in future research, may be by other researchers as well.

#### Laboratory sample collection, storage and transportation

1. From each eligible individual 5ml of venous blood will be collected at baseline and at the end of the intervention.
2. The serum will be separated from whole blood by collection tube
3. The serum will be transported to Nunc tube for storage and transportation
4. The serum will be stored in deep freezer at below -80°C until transportation and analysis
5. Collected samples will be transported to Bahir Dar University Teaching Hospital department of laboratory by using vaccine carrier
6. Finally, the laboratory measurement will be done by those special trained personnel at Bahir Dar University Teaching Hospital department of laboratory.

### Statistical analysis

The analysis will follow modified intention-to-treat principles (based on group randomized to and using all available data but without being able to directly assess the impact of the intervention being offered to those for whom we do not have follow-up data and where their outcome data are not missing at random). Descriptive statistics means (standard deviations), medians (interquartile ranges), frequencies (relative frequencies) will be used to summarize participant’ characteristics and study outcomes. Epi Data (double entry) will be used to enter the quantitative data and it will be analyzed using Stata 13.1 or later versions, with p<0.05 considered significant. Generalized linear mixed models (GLMM) will be used to assess the impact of the intervention on the change of metabolic syndrome components, as well as on the knowledge, attitudes and practice of participants on healthy lifestyle. The metabolic syndrome indicators (Obesity level, BP, FG, HDL, and TGL), knowledge, attitudes, and practices towards lifestyle and cardiovascular risk factors will be the outcome variables, and age, sex, marital status, educational status, work experience, working hours per day, monthly income, religion, health condition, and level of KAP will be the independent variables of the study.

**Fig 1:**
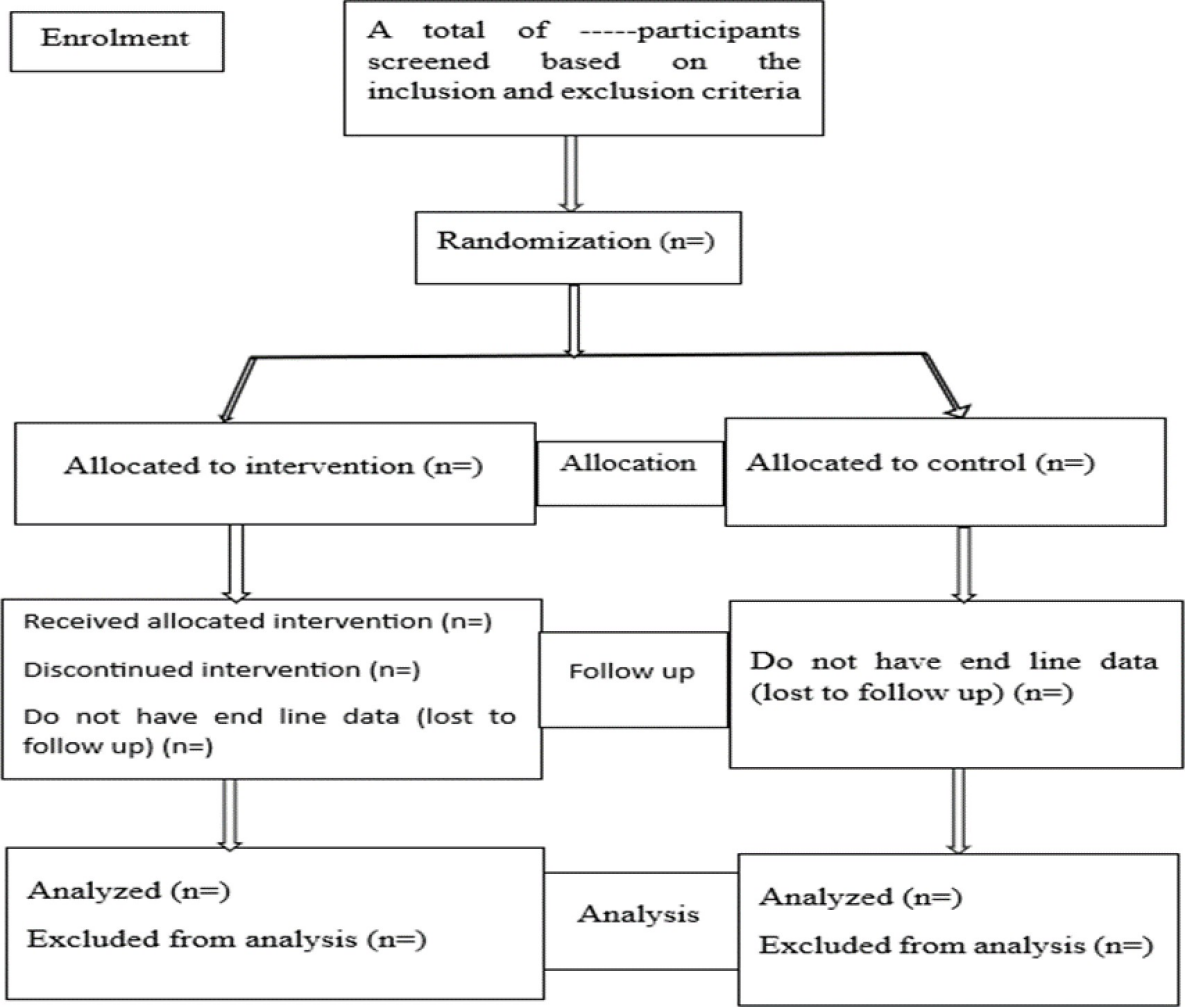
Trial flow diagram for the effectiveness of an education intervention promoting healthy lifestyle to reduce risk factors for metabolic syndrome, among office workers in Ethiopia.

All items in the CONSORT statement will be followed when reporting, and a CONSORT flow diagram will be included to indicate participant flow and, if available, the causes of exclusion and loss to follow-up (**Fig 1)**. The effectiveness of the intervention will be assessed using differences in changes in the metabolic syndrome indicators (Obesity level, BP, FG, HDL, and TGL) between groups from baseline to nine months. As well as, by assessing knowledge, attitudes, and practices towards lifestyle and cardiovascular risk factors before and after the intervention as well as between the control and intervention groups.

#### Qualitative data analysis

Thematic content analysis will be conducted. The Consolidated Framework for Implementation Research (CFIR) model will be used to analyze and present the data. First, the researcher will read the transcripts multiple times to become familiar with the data. Second, the data will be explored and examined for a pattern or repeated ideas that emerge from the data. Third, cods will be established to categorize the data. Fourth, data coding will be done line by line. Fifth, the codes will be linked into cohesive overarching sub-themes and then under main themes. Sixth, data reduction will be conducted. Finally, the researcher will write and interpret the results as per the themes.

### Ethical considerations and declarations

The Australian National University Human Ethics Committee and College of Medicine and Health Sciences Bahir Dar University Institutional Review Board approved this study 2022/845 and 792/2023 respectively. It is also registered with the Australian New Zealand Clinical Trials Registry (ACTRN12623000409673p).

Any important protocol modifications will be communicated to ethical review board and trial registry.

### The status and timeline of the study

Unique Protocol ID: 2022/845 and 792/2023 in Australian National University Human Ethics Committee and College of Medicine and Health Sciences Bahir Dar University Institutional Review Board respectively.

Australian New Zealand Clinical Trials Registry: ACTRN12623000409673p

Registration date: April 24, 2023

The date recruitment began: November 20^th^, 2023.

Approximate date when recruitment will be completed: December 29, 2023 [anticipated].

### Study timeline

The project’s enrollment, intervention, and assessment schedules are all well-established.

**Fig 2:**
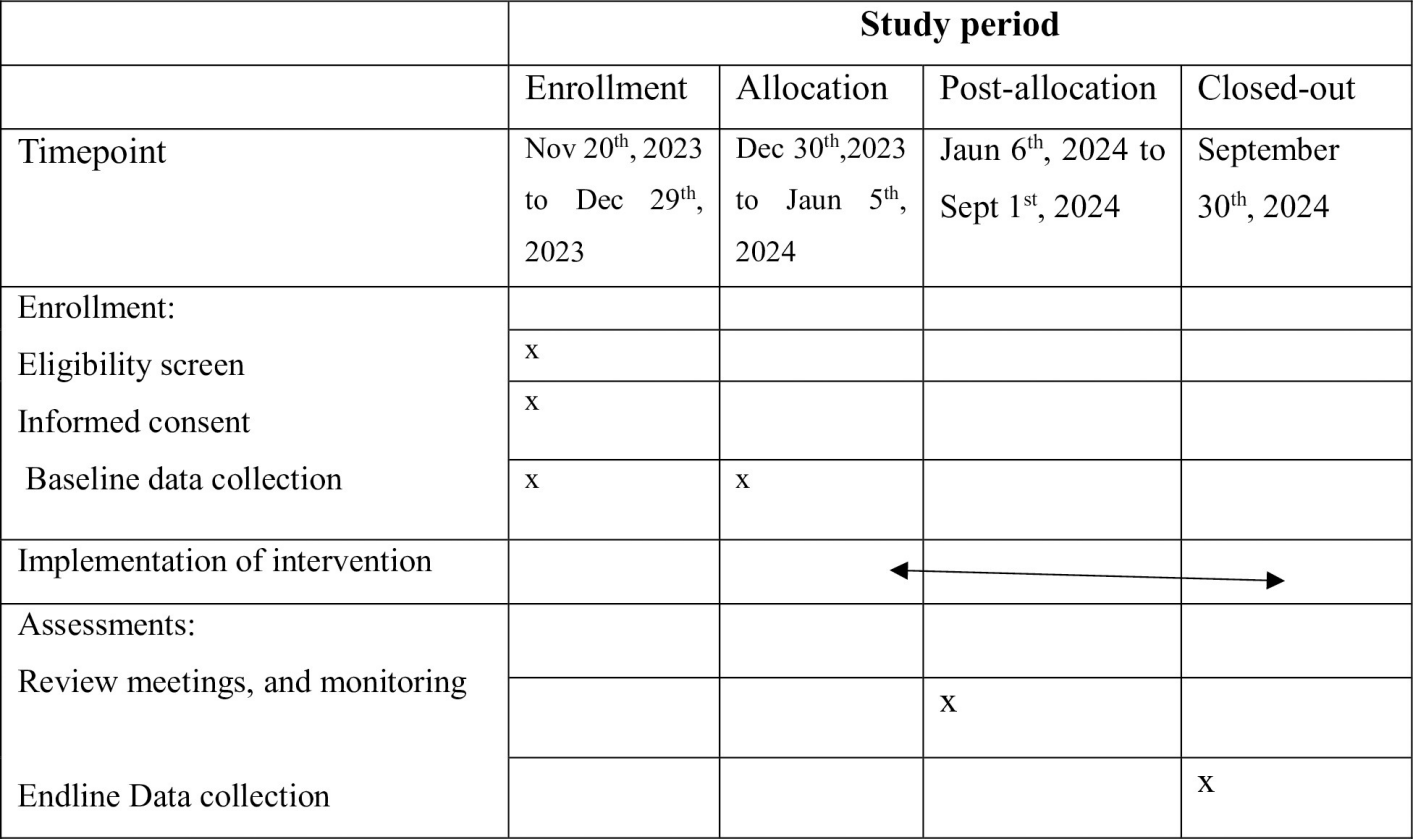
Timeline of recommended content for the schedule of enrolment, interventions, and assessments. The enrollment started in November 2023, and the project will be closed in September 2024.

The enrollment started in November 2023, and the project will be closed in September 2024 (**Fig 2)**.

## Discussion

This trial study will provide information on the effectiveness, feasibility, and acceptability of an education intervention promoting healthy lifestyle to reduce risk factors for metabolic syndrome, among office workers in Ethiopia, where the burden of metabolic syndrome is high among office workers [18, 19]. Additionally, it will offer evidence on the level of knowledge, attitude and practice towards lifestyle and cardiovascular risk factors among office workers in Ethiopia. It will also address context-related barriers to implementing a healthy lifestyle and the intervention’s cost-effectiveness. One-on-one based education will be applied to deliver the intervention, which will enhance outcomes and adherence by strengthening relationships with providers and creating a more intimate environment in which participants can talk openly about difficult themes [39]. Review meetings will also be held at the three and six-month mark. This will allow for any misunderstandings to be cleared up, the possibility of additional assessments, and the modification of intervention components and this will maximize the effectiveness of interventions [40]. The intervention of this trial study will address a wide range of topics (diet, physical activity, and psychological aspects), be conducted over a relatively long period of time (9 months), and be delivered by experts in health promotion. This will provide the participants with the chance to obtain comprehensive information, ample time to change their behaviors, sound knowledge, and the capacity to adopt a healthy lifestyle [41, 42]. A process evaluation will also be undertaken to identify context-specific delivery factors, facilitators and barriers to implementation.

The limitation of this trial study is that the intervention is not designed based on the needs of each participant and therefore, it may not be tailored for each individual.

### Dissemination of findings

The final result of the study will be submitted to both the Amhara public health institute and the district offices of banks in Bahir Dar city. Each participant will also receive the results through their previously registered information, which will be kept separate from the other research data. The results of the study will also be submitted for publication in scholarly journals and will be used to prepare thesis. Data will be securely stored on password-protected computers in the National Centre for Epidemiology and Population Health at the Australian National University. All research data will be kept for at least five years after publications resulting from the study and securely stored.

## Supporting information

S1 File SPIRIT 2013 checklist: Recommended items to address in a clinical trial protocol and related documents. (DOCX)

S2 File Self-report format to evaluate the enactment of the participants to the recommended healthy lifestyle to reduce risk factors for metabolic syndrome, among office workers in Ethiopia, 2023. YES/NO.
(DOCX)

S3 File Checklist to evaluate the implementation fidelity of educational intervention for healthy lifestyle to reduce risk factors for metabolic syndrome, among office workers in Ethiopia, 2023. (DOCX)

S4 File Protocol for ethical approval. (RTF)

S5 File Participant information sheet for randomized control trial study. (DOCX)
