# Supplementary material for: The effectiveness, feasibility, and acceptability of an education intervention promoting healthy lifestyle to reduce risk factors for metabolic syndrome, among office workers in Ethiopia: A protocol for a randomized control trial study
Source: PLoS One. 2024 Aug 30;19(8):e0307659. doi: 10.1371/journal.pone.0307659 (PMC11364252; doi:10.1371/journal.pone.0307659)
Supplement: S2 File — YES/NO. (DOCX) [file pone.0307659.s002.docx]

**Part I: Self-report format to evaluate the enactment of the participants to the recommended healthy lifestyle to reduce risk factors for metabolic syndrome, among office workers in Ethiopia, 2023. YES/NO**

| **S.no** | **Activities** | **Month 1** | | | | **Month 2** | | | | **Month3** | | | | **Month 4** | | | |
| --- | --- | --- | --- | --- | --- | --- | --- | --- | --- | --- | --- | --- | --- | --- | --- | --- | --- |
|  |  | **W1** | **W2** | **W3** | **W4** | **W1** | **W2** | **W3** | **W4** | **W1** | **W2** | **W3** | **W4** | **W1** | **W2** | **W3** | **W4** |
| **1** | Have you maintained the healthy diet recommendations, in the last week? |  |  |  |  |  |  |  |  |  |  |  |  |  |  |  |  |
| **2** | Have you done physical exercise as per the recommendations of the trainer in the week? |  |  |  |  |  |  |  |  |  |  |  |  |  |  |  |  |
| **3** | If you are a smoker, have you applied the recommended techniques to stop smoking in the last week? If you are not a smoker skip this part. |  |  |  |  |  |  |  |  |  |  |  |  |  |  |  |  |
| **4** | If you are alcohol consumer, have you used the recommended means to avoid alcohol consumption in the last week? If you are not alcohol user skip this section. |  |  |  |  |  |  |  |  |  |  |  |  |  |  |  |  |
| **5** | If you have faced a stressful event in the last week, have you used the recommended techniques to manage the stress? If you are not faced stress skip this section. |  |  |  |  |  |  |  |  |  |  |  |  |  |  |  |  |
| **6** | Have you read and responded to the text messages that was sent from the researcher in the last two weeks? |  | |  | |  | |  | |  | |  | |  | |  | |

***W=Week**

**What are the barriers that hindered you to apply the recommended healthy lifestyle? Please list the barriers that you faced.**

**------------------------------------------------------------------------------------------------------------------------------------------------------------------------------------------------------------------------------------------------------------------------------------------------------------------------------------------------------------------------------------------------------------------------------------------------------------------------------------------------------------------------------------------------------------------------------------------------------------------------------------------------------------------------------------------------------------------------------------------------------------------------------------------------------------------------------------------------------------------------------------------------------------------------------------------------------------------------------------------------------------------------------------------------------------------------------------------------------------------------------------------------------------------------------------------------------------------------------------------------------------------------------------------------------------------------------------------------------------------------------------------------------------------------------------------------------------------------------------------------------------------------------------------------------------------------------------------------------------------------------------------------------------------------------------------------------------------------------------------------------------------------------------------------------------------------------------------------------------------------------------------------------------------------------------------------------------------------------------------------------------------------------------------------------------------------------------------------------------------------------------------------------------------------------------------------------------------------------------------------------------------------------------------------------------------------------------------------------------------------------------------------------------------------------------------------------------------------------------------------------------------------------------------------------------------------------------------------------------------------------------------------------------------------------------------------------------------------------------------------------------------------------------------------------------------------------------------------------------------------------------------------------------------------------------------------------------------------------------------------------------------------------------------------------------------------------------------------------------------------------------------------------------------------------------------------------------------------------------------------------------------------------------------------------------------------------------------------------------------------------------------------------------------------------------------------------------------------------------------------------------------------------------------------------------------------------------------------------------------------------------------------------------------------------------------------------------------------------------------------------------------------------------------------------------------------------------------------------------------------------------------------------------------------------------------------------------------------------------------------------------------------------------------------------------------------------------------------------------------------------------------------------------------------------------------------------------------------------------------------------------------------------------------------------------------------------------------------------------------------------------------------------------------------------------------------------------------------------------------------------------------------------------------------------------------------------------------------------------------------------------------------------------------------------------------------------------------------------------------------------------------------------------------------------------------------------------------------------------------------------------------------------------------------------------------------------------------------------------------------------------------------------------------------------------------------------------------------------------------------------------------------------------------------------------------------------------------------------------------------------------------------------------------------------------------------------------------------------------------------------------------------------------------------------------------------------------------------------------------------------------------------------------------------------------------------------------------------------------------------------------------------------------------------------------------------------------------------------------------------------------------------------------------------------------------------------------------------------------------------------------------------------------------------------------------------------------------------------------------------------------------------------------------------------------------------------------------------------------------------------------------------------------------------------------------------------------------------------------------------------------------------------------------------------------------------------------------------------------------------------------------------------------------------------------------------------------------------------------------------------------------------------------------------------------------------------**

**Part II: Self-report format to evaluate the enactment of the participants to the recommended healthy lifestyle** **to reduce risk factors for metabolic syndrome, among office workers in Ethiopia, 2023. YES/NO**

| **Sno** | **Activities** | **Month 5** | | | | **Month 6** | | | | **Month 7** | | | | **Month 8** | | | | **Month 9** | | | |
| --- | --- | --- | --- | --- | --- | --- | --- | --- | --- | --- | --- | --- | --- | --- | --- | --- | --- | --- | --- | --- | --- |
|  |  | **W 1** | **W2** | **W3** | **W4** | **W1** | **W2** | **W3** | **W4** | **W1** | **W2** | **W3** | **W4** | **W1** | **W2** | **W3** | **W4** | **W1** | **W2** | **W3** | **W4** |
| **1** | Have you maintained the healthy diet recommendations, in the last week? |  |  |  |  |  |  |  |  |  |  |  |  |  |  |  |  |  |  |  |  |
| **2** | Have you done physical exercise as per the recommendations of the trainer in the last week? |  |  |  |  |  |  |  |  |  |  |  |  |  |  |  |  |  |  |  |  |
| **3** | If you are a smoker, have you applied the recommended techniques to stop smoking in the last week? If you are not a smoker skip this section. |  |  |  |  |  |  |  |  |  |  |  |  |  |  |  |  |  |  |  |  |
| **4** | If you are alcohol consumer, have you used the recommended means to avoid alcohol consumption in the last week? If you are not alcohol user skip this section. |  |  |  |  |  |  |  |  |  |  |  |  |  |  |  |  |  |  |  |  |
| **5** | If you have faced a stressful event in the last week, have you used the recommended techniques to manage the stress? If you are not faced stress skip this section. |  |  |  |  |  |  |  |  |  |  |  |  |  |  |  |  |  |  |  |  |
| **6** | Have you read and responded to the text messages that was sent from the researcher in the last two weeks? |  | |  | |  | |  | |  | |  | |  | |  | |  | |  | |

***W=Week**

**What are the barriers that hindered you to apply the recommended healthy lifestyle? Please list the barriers that you faced.**

**--------------------------------------------------------------------------------------------------------------------------------------------------------------------------------------------------------------------------------------------------------------------------------------------------------------------------------------------------------------------------------------------------------------------------------------------------------------------------------------------------------------------------------------------------------------------------------------------------------------------------------------------------------------------------------------------------------------------------------------------------------------------------------------------------------------------------------------------------------------------------------------------------------------------------------------------------------------------------------------------------------------------------------------------------------------------------------------------------------------------------------------------------------------------------------------------------------------------------------------------------------------------------------------------------------------------------------------------------------------------------------------------------------------------------------------------------------------------------------------------------------------------------------------------------------------------------------------------------------------------------------------------------------------------------------------------------------------------------------------------------------------------------------------------------------------------------------------------------------------------------------------------------------------------------------------------------------------------------------------------------------------------------------------------------------------------------------------------------------------------------------------------------------------------------------------------------------------------------------------------------------------------------------------------------------------------------------------------------------------------------------------------------------------------------------------------------------------------------------------------------------------------------------------------------------------------------------------------------------------------------------------------------------------------------------------------------------------------------------------------------------------------------------------------------------------------------------------------------------------------------------------------------------------------------------------------------------------------------------------------------------------------------------------------------------------------------------------------------------------------------------------------------------------------------------------------------------------------------------------------------------------------------------------------------------------------------------------------------------------------------------------------------------------------------------------------------------------------------------------------------------------------------------------------------------------------------------------------------------------------------------------------------------------------------------------------------------------------------------------------------------------------------------------------------------------------------------------------------------------------------------------------------------------------------------------------------------------------------------------------------------------------------------------------------------------------------------------------------------------------------------------------------------------------------------------------------------------------------------------------------------------------------------------------------------------------------------------------------------------------------------------------------------------------------------------------------------------------------------------------------------------------------------------------------------------------------------------------------------------------------------------------------------------------------------------------------------------------------------------------------------------------------------------------------------------------.**
