# Supplementary material for: The effectiveness, feasibility, and acceptability of an education intervention promoting healthy lifestyle to reduce risk factors for metabolic syndrome, among office workers in Ethiopia: A protocol for a randomized control trial study
Source: PLoS One. 2024 Aug 30;19(8):e0307659. doi: 10.1371/journal.pone.0307659 (PMC11364252; doi:10.1371/journal.pone.0307659)
Supplement: S3 File — (DOCX) [file pone.0307659.s003.docx]

**Checklist to evaluate the implementation fidelity of educational intervention for healthy lifestyle to reduce risk factors for metabolic syndrome, among office workers in Ethiopia, 2023.**

| **Objective** | **Program**  **Component** | **Exposure** | | **Specific Features** | **Adherence**  **Yes/No** | **Quality:**  **1 = Low (confusing),**  **3 = Medium,**  **5 = High (clear)** | **Responsiveness:**  **1 = Low (unengaged),**  **3 = Medium,**  **5 = High (engaged)** |
| --- | --- | --- | --- | --- | --- | --- | --- |
|  |  | **Planned** | **Actual** |  |  |  |  |
| -To describe about the metabolic syndrome  -To reducing the rate of non-communicable diseases | Definition of metabolic syndrome,  risk factors and prevention. | **15 minutes** |  | Metabolic syndrome (MetS) is a collection of disorders that includes:   - Abdominal obesity, - High blood pressure, - Impaired fasting glucose, - High triglyceride levels, and - Low HDL cholesterol levels   Prevention of metabolic syndrome is a lifelong commitment to a healthy lifestyle.  **A healthy lifestyle includes:**   - Getting at least 30 minutes of physical activity most days, - Eating plenty of vegetables, fruits, lean protein and whole grains. - Limiting saturated fat and salt in the diet. - Avoidance of harmful alcohol consumption, free from smoking and managing stress. |  |  |  |
| -To improve knowledge, attitude, and behaviours regarding healthy diets and manage  metabolic syndrome  -To motivating the subjects to modify their unhealthy behaviours  -To reducing the rate of non-communicable diseases | Healthy eating | **15 minutes** |  | -Participants will be encouraged to develop healthy eating habits by increasing their intake of fresh fruits and vegetables at least 400 g per day, or five portions of fruit and vegetables excluding (starchy roots like potatoes, sweet potatoes, and cassava), as well as whole grains like unprocessed maize, millet, oats, wheat, and brown rice, legumes like lentils and beans, nuts, fish, white meat, and low-fat dairy products.  -Moreover, the participants will be advised not to use saturated fats such as solid and animal oils and butter and replace them with liquid vegetable oils.  -Further, the participants will be motivated to reduce the consumption of fried food, fast food, and instead consume a diet with a restricted amount of sugar, sweetmeat, starch, and red meat products.  -Additionally, emphasis will be placed on consuming no more than two teaspoons of salt daily, and people will be urged not to use the saltshaker when setting the table. |  |  |  |
| -To improve knowledge, attitude, and behaviours regarding appropriate physical activities and manage metabolic syndrome  -To motivating the subjects to modify their unhealthy behaviours  -To reducing the rate of non-communicable diseases | Physical activity | **15 minutes** |  | -Participants will encourage to do at least 150–300 minutes of moderate-intensity aerobic physical activity; or at least 75–150 minutes of vigorous-intensity aerobic physical activity; or an equivalent combination of moderate- and vigorous-intensity activity throughout the week.  -They will also be recommended to increase physical exercise, such as walking, cycling, brisk walking, or climbing stairs, while reducing sedentary lifestyles (watching TV, using the Internet, etc.).  -Workplace and household physical activities |  |  |  |
| -To improve knowledge and skills to manage Stresses  -To motivating the subjects to modify their unhealthy behaviours  -To reducing the rate of non-communicable diseases | Stress management | **10 minutes** |  | -Accepting changes that are uncontrollable  -Keeping work and rest in proportion  -Daily 15-20 minutes of relaxation  -Moving to overcome fatigue |  |  |  |
| -To explain how to quit smoking  -To motivating the subjects to modify their unhealthy behaviours  -To reducing the rate of non-communicable diseases | Smoking | **10 minutes** |  | Provide brief information for the participants to develop a quit plan using the **STAR** method:  -Set a quit date, ideally within 2 weeks.  -Tell family and friends about quitting and ask for support.  -Anticipate challenges in the quit attempt.  -Remove tobacco products from personal environment and make home smoke-free |  |  |  |
| -To explain techniques to avoid harmful alcohol consumption  -To motivating the subjects to modify their unhealthy behaviours  -To reducing the rate of non-communicable diseases | Harmful alcohol consumption | **10 minutes** |  | The following information will be provided to participants in order to prevent harmful alcohol consumption:  -Overall, abstaining from alcohol is the best strategy to reduce your risk of health problems.  -If you choose to consume alcohol, remember that "less is better."  -Aim for no more than two units each day, and abstain from alcohol on at least two days of the week.  -Do not drink alcohol for “health” reasons. |  |  |  |
| -To discuss on the limitations and challenges as well as potential solutions to meet the recommendations of the intervention | Challenges and solutions to meet the recommendations of the intervention to healthy lifestyle | **15 minutes** |  | Cultural, social, workplace, individual and other factors that will affect to meet the recommended interventions and potential solutions to the factors will be identified by participants, education providers and researcher. |  |  |  |
| Delivering reminder text messages | Recommended healthy lifestyles | **18 text messages** |  | Adherence to the recommended healthy diets, physical exercise, quit smoking, avoidance of harmful alcohol consumption, stress management and use of strategies to solve problems to adhere the healthy life style as well as filing of self-report format every month.  -They will be asked if they keep track of their physical activity records, and they will be urged to reply to messages by saying “Yes” or “No”, ask questions, and look for advice. |  |  |  |
| -To increase health literacy, skills and motivation among the participants.  -To promote and encourage the individuals, and increase the memorise and adherence  -To share their experiences regarding lifestyle modification | Review meetings | **Twice** |  | -Revision of main points about healthy lifestyle  -Invite them to share their experiences to the researcher |  |  |  |
