# Supplementary material for: The effectiveness, feasibility, and acceptability of an education intervention promoting healthy lifestyle to reduce risk factors for metabolic syndrome, among office workers in Ethiopia: A protocol for a randomized control trial study
Source: PLoS One. 2024 Aug 30;19(8):e0307659. doi: 10.1371/journal.pone.0307659 (PMC11364252; doi:10.1371/journal.pone.0307659)
Supplement: S4 File — (RTF) [file pone.0307659.s004.rtf]

Created by: u7317922 
Record number: 13974 
Protocol type: Full Ethical Review 
Protocol number: 2022/845 
Date entered: 22/03/2023 
Ethics program type: Postgraduate 
Requested start date: 22/03/2023 
Requested end date: 22/03/2025 

Protocol title: The effectiveness, feasibility, and acceptability of an education intervention promoting healthy lifestyle to reduce risk factors for metabolic syndrome, among office workers in Ethiopia: A randomized control trial. 
 
Investigators 

	Name 
	Role 
	Department 
	
	Bogale, Sitotaw	Primary investigator	Department of Global Health, CHM National Centre for Epidemiology and Population Health, ANU	
	Gray, Darren	Supervisor	Department of Global Health, CHM National Centre for Epidemiology and Population Health, ANU	
	Kelly, Matthew	Lead supervisor	Department of Global Health, CHM National Centre for Epidemiology and Population Health, ANU	
	Sarma, Haribondhu	Supervisor	Department of Global Health, CHM National Centre for Epidemiology and Population Health, ANU	
				

Investigators Detailed 
Name:  Bogale, Sitotaw            Role:  Primary investigator 
Expertise:  Sitotaw Bogale
I have MSc in Adult Health Nursing, and have multidisciplinary professional experiences in teaching, supervising students, community services and have substantial experience in the design, conduct and publication of research for more than 7 years in different Ethiopian universities. I have conducted over 16 research studies, 11 of which have been published in scholarly journals. My research areas of expertise were non-communicable diseases, women's health, and mental health.  Also, I won and completed successfully the following research projects; 1) Metabolic syndrome among people of Mizan-Aman Town, South West Ethiopia; 2) Postpartum depression in south-west Ethiopia. Due of my efforts to research, teaching, and community services, Bahir Dar University promoted me to assistant professor. Additionally, I have taken a training on research integrity from Australian National University. Since I have conducted similar studies with my current project, I have experience in how to approach participants, how to collect data from study participants, and how to collect, store, and transport laboratory samples for analysis. The study will take place in Ethiopia, particularly in the city of Bahir Dar, where I currently live and work. Amharic will be the study's primary language because it is the first language of both the researcher and the participants.
Name:  Gray, Darren            Role:  Supervisor 
Expertise:  Professor Darren Gray
Professor Darren has research experiences in the fields of epidemiology, health promotion, public health, infectious diseases, medical parasitology, and Microbiology. He has also extensive experience in managing organizations and teams, conducting and leading fieldworks, and supervising students.
Name:  Kelly, Matthew            Role:  Lead supervisor 
Expertise:  Dr Matthew Kelly
Dr Matthew Kelly is an expert in the field of epidemiology, public health, public health nutrition, and major global burdens of disease. He is also knowledgeable in research areas of mortality, social determinants of health, and health services. Additionally, he has field work experiences and supervised numerous both domestic and foreign, PhD students.
 
Name:  Sarma, Haribondhu            Role:  Supervisor 
Expertise:  Dr Haribondhu Sarma
Dr Sarma has an experience in the research fields of implementation science and evaluation, nutritional epidemiology, health promotion, public nutrition intervention, public health and health services, social epidemiology, and social determinants of health. He has also experienced of supervising students and conducting field works in international settings.
External Investigators 

	Name 
	Role 
	Institution 
	
				
Departments 

	Primary 	Department 	Faculty 	
	Yes	Department of Global Health	CHM National Centre for Epidemiology and Population Health	

   Project Questions Detailed 
Description of Project  
Describe the research project in terms easily understood by a lay reader, using simple and non-technical language. Non-communicable diseases are the number one cause of deaths globally [1]. Metabolic syndrome is a collection of disorders that comprise: obesity, insulin resistance, glucose intolerance, impaired regulation of body fat and high blood pressure [2]. Metabolic syndrome is the primary cause of cardiovascular disease, and increases risk of developing type 2 diabetes, two of the four most common non-communicable diseases [3].
Metabolic syndrome has become a major health threat for the modern world. More than one billion people in the globe are now affected by metabolic syndrome and the condition plays a substantial role in the blowout of diseases like type 2 diabetes, coronary diseases, stroke, and other chronicity [4]. In addition to increasing the risk of acquiring non-communicable diseases, it also involves expensive medical care [5]. According to some research, individuals with metabolic risk factors incur healthcare expenses that are up to 20% more than those of patients without risk factors [6].
In general, the prevalence of metabolic syndrome is increasing globally as a result of epidemiological changes, and the current trend cannot be maintained unless efforts are made to change behavioral risk factors that are causing the prevalence of metabolic syndrome. This can be done by developing and implementing appropriate interventions that can bring the behavior change [7]. Although metabolic syndrome is becoming more common in developing countries like Ethiopia, there is still a lack of public awareness as the condition is new to the region [8, 9]. And, also there is a lack of locally relevant interventions so far. Therefore, it suggests that Ethiopia is a prime candidate for intervention to make change on the spread of metabolic syndrome.
An educational intervention for a healthy lifestyle is a method of enabling people to recognize the variables that affect individual-social and mental well-being and to make the best choices for adopting healthy behaviors [10]. It helps people adopt and maintain behaviors connected to a lifestyle that promotes health by increasing knowledge, self-efficacy, and bringing about behavioral change [11, 12]. However, educational interventions for healthy lifestyle have not been developed and tested for effectiveness, feasibility and acceptability in Ethiopia. The barriers and enablers to adopt the healthy lifestyle strategies in Ethiopia are unexplored. 
Thus, this study will provide evidence about the barriers and opportunities to adopt healthy lifestyle strategies and the effectiveness, feasibility, acceptability of lifestyle intervention on metabolic syndrome, the level of knowledge, attitudes and practice towards lifestyle and cardiovascular risk factors among office workers with metabolic syndrome in Ethiopia. Consequently, the evidence will be used for policy makers to scale up these management strategies and for stakeholders, as an input to plan effective metabolic syndrome management strategy, and will also be used as a reference for other randomized control trial study.  Moreover, it will be used as evidence for professionals to apply this intervention for risky groups. 
Location of Data Collection  
Australia No 
Overseas Yes 
Provide country / area where data collection will be conducted 
The study will be conducted in the Bahir Dar city, Ethiopia. Bahir Dar is located 565 km Northwest of Addis Ababa and it is the capital city of Amhara regional state. 

Aims of the Project  
List the hypothesis and objectives of your research project. 
The aim of this project is to develop and test the effectiveness, feasibility, and acceptability of an education intervention promoting healthy lifestyle to reduce risk factors for metabolic syndrome, among office workers in Ethiopia
The hypotheses of project are;  
1. An educational lifestyle intervention will be effective, feasible, and acceptable in metabolic syndrome management for bank workers in Ethiopia. 
2. There will be different levels of knowledge, attitudes and practices towards lifestyle and cardiovascular risk factors between pre and post intervention, and after the intervention between control and intervention groups, among bank workers with metabolic syndrome in Ethiopia.
3. There will be barriers and enablers that hinder or facilitate the implementation of healthy lifestyle strategies and different lifestyle experiences among employed adults in Ethiopia. 
The objectives of this research are to;
1.	Explore the barriers and enablers to implement healthy lifestyle strategies, as well as the lifestyle experiences of employed adults in Ethiopia. 
2.	To develop and test the effectiveness of a lifestyle education intervention on metabolic syndrome management among bank employees in Ethiopia.
3.	To assess and compare the levels of knowledge, attitudes, and practices towards lifestyle and cardiovascular risk factors before and after the intervention as well as between the control and intervention groups among bank employees with metabolic syndrome in Ethiopia. 
4.	To assess the feasibility, and acceptability of an education intervention for healthy lifestyle on metabolic syndrome management among bank employees in Ethiopia.
5.	To evaluate the cost-benefit of the educational intervention promoting healthy lifestyle to reduce risk factors for metabolic syndrome among bank employees in Ethiopia.
6.	To evaluate the process of education intervention promoting healthy lifestyle to reduce risk factors for metabolic syndrome for bank employees in Ethiopia.


Background Review  
Provide evidence that the proposed research is based on the relevant literature and indicate how the research draws from and will contribute to that literature. Highlight any ethical issues that have been considered and addressed in the literature. 
The education intervention for healthy lifestyle is recommended by the studies as it is one of the successful interventions in changing behaviors that are connected to a healthy lifestyle and its components. An education intervention for healthy lifestyle is an intervention that help people adopt and maintain behaviors connected to a lifestyle that promotes health by increasing knowledge, self-efficacy, and behavior changes on diet and physical activity. A prospective, longitudinal, nonequivalent pretest-posttest comparison group design study conducted in San Jose, Costa Rica and Chiapas, Mexico to evaluate the impact of education intervention for healthy lifestyle to reduce cardiovascular diseases confirmed that there was a significant improvement of behavior change in the intervention group [13]. 
However, the contexts that can affect the effectiveness of education intervention for healthy lifestyle have not been explained in Ethiopia, or anywhere in sub-Saharan Africa as far as my knowledge. The barriers and opportunities to apply the healthy lifestyle strategies in Ethiopia are unexplored. The effectiveness, feasibility and acceptability are not tested in Ethiopia. The level of knowledge, attitudes and practice towards lifestyle and cardiovascular risk factors among bank workers with metabolic syndrome in Ethiopia, are not judged. 
Thus, this study will provide evidence about the effectiveness of a workplace educational intervention for the management of metabolic syndrome and its components, the barriers and opportunities to implement healthy lifestyle strategies and the feasibility, acceptability and effect of lifestyle intervention on the metabolic syndrome, the level of knowledge, attitudes and practice towards lifestyle and cardiovascular risk factors among bank employees with metabolic syndrome in Ethiopia. Consequently, the evidence will be used as in input for literatures. There was no any ethical problem that have been considered and addressed in the literature.
Methodology  
In language appropriate for a lay reader, explain why the methodological approach minimizes the risk to participants. (For surveys, include justification of the sample size). This research project will have two parts. The first part is qualitative study that will be conducted among employed adults in Bahir Dar town, Ethiopia, to explore the enablers and barriers to adopting healthy lifestyle strategies, and their personal lifestyle experiences, as well as possible suggestions regarding the solutions for barriers. The second part is a randomized control trial to develop and test the effectiveness, feasibility, and acceptability of an education intervention promoting healthy lifestyle to reduce risk factors for metabolic syndrome, among office workers, and to assess and compare the levels of knowledge, attitudes, and practices towards lifestyle and cardiovascular risk factors before and after the intervention as well as between the control and intervention groups among bank workers with metabolic syndrome in Ethiopia. 
1. For qualitative study
This qualitative study will be carried out in Bahir Dar, Ethiopia, on employed adults (bank employees, teachers, lawyers, health professionals, tele workers, and journalists who will be recruited in the manner described in the participant details section below), to explore the enablers and barriers to adopting healthy lifestyle strategies, and their personal lifestyle experiences, as well as possible suggestions regarding the solutions for barriers. The possible risks due to participating in this qualitative study include lost time and embarrassment while discussing an unhealthy way of life or habit. We will minimize loss of time by conducting the interviews in the shortest time as much as possible (we estimated that it would take around an hour), and by using the convenient time chosen by the participant. Also, we will minimize embarrassment by doing one-on-one interviews and maintaining privacy. We will reassure participants that they can chose not to answer any questions they are uncomfortable with, and that they can end the interview at any time. 
2. For randomized control trial (RCT)
Bank employees will be recruited and screened based on their level of obesity and blood pressure, and those who meet at least one of the two criteria (obesity or high blood pressure) will be included in the trial and have biomarkers tested (Fasting blood glucose level, High density lipoprotein cholesterol, low density lipoprotein cholesterol, and triglyceride). The individuals who meet the study's inclusion criteria (described in the section on inclusion criteria) will be included in the trial and a pre-assessed and validated lifestyle education intervention will be implemented in collaboration with experts. 
A randomized control trial study with a parallel design will be carried out to develop and test the effectiveness, feasibility, and acceptability of a lifestyle intervention in reducing the risk factors of  metabolic syndrome, and assess and compare the levels of knowledge, attitudes, and practices towards lifestyle and cardiovascular risk factors between pre and post intervention,  and after the intervention between the control and intervention groups, among bank workers with metabolic syndrome in Ethiopia. The sample size for this randomized control trial study was determined under the assumptions and using the formula of the sample size calculation for randomized control trial studies with continuous outcome variables [14]. The means and standard deviations of systolic blood pressure were taken from a study with a similar design that was conducted in Ethiopia, because it is the most common indicator of metabolic syndrome among bank employees in Bahir Dar town among other indicators [15]. A power of 80%, a 1:1 ratio, and a 95% confidence interval were used for calculation. 
Then the sample size was calculated as follows;   
n1= (ó12 +ó22) [ Z1-á /2+ Z 1-â]2  
           (M1-M2)2   
n1= (15.02 + 16.32) [1.96+0.84]2    = (225+265.69) [2.8]2     = (490.69) (7.84)  =94 
     (107.8 -114.2)2                                 6.42                                     40.96                                                                                   
n1=94 and n2=n1*1=94, Therefore, N= n1+n2=188
where,
m1=mean of systolic blood pressure for the intervention group 
m2= mean of systolic blood pressure for the control group
ó1=standard deviation of systolic blood pressure for the intervention group
ó2=standard deviation of systolic blood pressure for the control group
n1 = sample size for group 1
n2 = sample size for group 2
á = probability of type I error (usually 0.05)
â = probability of type II error (usually 0.2)
z = critical Z value for a given á or â
N= Total sample size
By taking into account a 20% attrition rate, 226 total individuals or 113 individuals per group will be recruited for the study. 
Additionally, self-administered questionnaires, physical measurements (waist circumference and blood pressure), and 5 ml of blood sample under strict infection control procedures will be used in this randomized control trial study to collect data. Therefore, Participants will not be at risk, with the exception of some discomforts due to concerns about social stigma related with unhealthy lifestyle (smoking, drinking, and obesity). Some may also find it uncomfortable to give blood samples and undergo physical measures. Even if someone wants to participate in the study, they will not be included if they refuse to provide blood sample or take physical measurements.  It will also be challenging to maintain perfect confidentiality because participants will be participating together in the intervention/training sessions. And it will be tough to conceal identities if several of them work in the same bank offices. 
The use of sensitive words relating to obesity, smoking, and drinking will be avoided during presentations, to prevent the happening of stigma. We will lessen discomforts related to drawing a blood sample and physical measurements by informing them that we will draw blood with a small size needle, follow strict infection control procedures, and perform physical measurements with trained data collectors who share the same gender as the participants. And group norms to protect co-workers' secrets will also be established before starting of education. All of this information will be included in the participants information sheet, and data collection and education will only begin if participants have agreed and given their approval.
 Provide the survey method, a list of the questions to be asked or an indicative sample of questions. These should give a good sense of the most intrusive/sensitive areas of questioning. 
1. For the qualitative study
The data will be collected through one-on one in-depth interviews using the Amharic language. The focus of the in-depth interview will be on participants understanding of the concept of a healthy lifestyle, sources of information about healthy lifestyle, barriers to implementing a healthy lifestyle, enablers to implement a healthy lifestyle, and their personal lifestyle experiences as well as possible suggestions regarding the solutions for barriers. Interviews will be recorded, transcribed and coded with NVivo12 software. The interview guideline is attached here as a supportive document.
2. For randomized control trial 
This randomized control trial will have intervention and control groups and the intervention will be given as follows; 
For intervention group: 
The intervention will consist of education about the concept of metabolic syndrome, risk factors, and prevention strategies, the significance of lifestyle modification such as exercise, a healthy diet, avoiding drinking alcohol that is harmful, quitting smoking, and stress management, as well as motivation for changing behavior. The WHO recommendation and the theory of planned behavior of change served as the foundation for its development, and each intervention component will be evaluated and validated by subject-matter experts. The education intervention will be delivered by experts on health promotion after the education package is assessed and validated by a nutritionist, physical therapist and psychologist. The education will be delivered in a one-on-one based at their office or at any other suitable places. Every two weeks, the researcher will send a text message to the participants as a reminder asking whether they have followed the suggested healthy diet, physical activity, and mechanisms of stress management, avoidance of harmful alcohol consumption and smoking. It will also enquire as to whether they recorded their actions using the provided self-report template. Throughout the intervention, the participants will be encouraged to respond to the messages, ask questions, and seek guidance. Reading materials (Handout) will also be given to each participant with the key messages of “avoid the three whites including: fat, sugar and salt, have a healthy diet, aerobic exercise, avoid sources of trans fats, avoid smoking, avoid unsafe intake of alcohol (≥ 2 drinks per day), manage your stress and avoid sitting for long time”. Additionally, a review meeting will be held at the 3rd and 6th month of the intervention. The primary investigator will supervise the education process.
For control group:  General health advice will be given in an individual form by the nursing professionals on physical exercising, diet, self-care and monitoring, according to the national guidelines recommended for non-pharmacological management for each component of the metabolic syndrome. An education package and healthy lifestyle booklets will also be provided for them at the end of the study.
For both groups: The baseline data about socio-demographic information (eg. age, sex, religion etc), metabolic syndrome status, knowledge, attitude and practice towards lifestyle and cardiovascular risk factors will be collected as follows from both groups before the intervention is delivered. 
Socio-demographic information will be collected through structured questionnaires, data about metabolic syndrome will be collected by measuring waist circumference, blood pressure, fast blood glucose level, total cholesterol, high density lipoprotein, low density lipoprotein, and triglycerides. Waist circumference will be measured with a tape measure at the approximate midpoint between the lower margin of the last palpable rib and the top of the iliac crest or at the abdomen's maximum extension. Blood pressure values will assess by appropriate size sphygmomanometer. Blood samples will be collected before breakfast by employing infection prevention procedures for the analysis of biochemical markers. 
Knowledge and attitudes towards lifestyle and cardiovascular risk factors will be assessed using a valid 43-item self-administered questionnaire (16). The questions will include address knowledge about coronary vascular disease and its risk factors, attitude towards coronary vascular disease and its risk factors. Practices of study participants on healthy lifestyle will be assessed using short form food frequency questionnaire (17).
Feasibility and acceptability will be measured by Feasibility of Intervention Measure (FIM), and Acceptability of Intervention Measure (AIM) (18), self-administered questionnaire respectively, at the 6th month of the intervention. The focus of the questions will be on feasibility and acceptability of the intervention. In addition to this, the acceptability of the intervention will also be assessed using Sekhons theoretical framework for health care interventions (19).  Every two weeks, a text message will be sent to the participants as a reminder. Whether they adhere to the recommended diet, physical exercise routine, and other intervention components will be covered in the text message's content. Additionally, they will be asked if they keep track of their physical activity records, and they will be urged to reply to messages by saying Yes or No, ask questions, and look for advice. Additionally, a review meeting will be held at the 3rd and 6th month of the intervention.
The data collection about metabolic syndrome and knowledge, attitudes and practice will be repeated at the 9th month of the intervention with the same method of the baseline. The data will be collected by trained nurses and laboratory technologist. 
What mechanisms do the researchers intend to implement to monitor the conduct and progress of the research project? For example: 
How often will the researcher be in touch with the supervisor?
Is data collection going as expected? If not, what will the researcher do?
Is the recruitment process effective?
How will the researcher monitor participants' willingness to continue participation in the research project, particularly when the research is ongoing? 
Every two weeks, there will be scheduled meetings with the supervisors. They will be updated on the research progress. If the process of data collection is not going as planned, I will attempt to fix it by discussing with the supervisors. To make recruiting effective and to increase participant willingness, the objectives of the study, inclusion and exclusion criteria, ethical considerations of the study, the advantages of the study and their rights even to stop in the middle of the procedure will be explained to participants. The participants will be informed that their participation in this study is very important for the success of the study and for paving the way for the development of the policy in this area. Additionally, they will be told that there is no risk except some discomforts to them in taking part in this study, and that all data obtained from them would be kept confidential via a password with no requirement that their identities be recorded, and results will also be communicated to them at the end of the study. They will also be told that even if they do not meet the requirements to participate in the study, they will still get booklets on living a healthy lifestyle and advice about seeking medical counsel if they exhibit any signs of metabolic syndrome. Finally, before any data is collected, signed consent will be obtained. 
Participants  
Provide details in relation to the potential participant pool, including: 
target participant group;
identification of potential participants;
initial contact method, and
recruitment method. 
1. For qualitative study 
The participants of this qualitative study will be employed adults in Bahir Dar town, and the key informants will be bank employees, teachers, lawyers, health professionals, tele workers and journalists. The compositions of the important key informants will be different ages, positions, health conditions, income, educational status, professions, marital status, religions, and gender. To select the key informants, the Amhara Public Health Institute will send a cooperative letter to banks, schools, courts, health institutions, tele and media offices. Then the manager of each office will give a permission to me as well as communicate with the staffs to cooperate with me. Once I have a permission to choose the key informants, I will select them purposively after asking them about their years of age, position, health conditions, income, educational status, marital status, and religions.  
                         
2. For randomized control trial 
The participants of this randomized control trial will be the employees of the banks in Bahir Dar, Ethiopia. The following methods will be applied to contact and recruit participants: The district headquarters of each bank will get an official letter from the Amhara Public Health Institution, after which letters will be issued to each branch from the head office and communications will be made between the managers of each branch and the bank employees. A meeting will be held with employees who have expressed interest in the study where we will explain the study aims and objectives and inclusion criteria. Then, after obtaining each participant's signed informed consent, the screening will proceed consecutively depending on the inclusion and exclusion criteria.

Proposed number of participants 
Number of participants in randomized control trial will be 226 individuals  
Provide details as to why these participants have been chosen? 
1. For qualitative 
Employed adults of Bahir Dar town are selected as key informants for this qualitative study because I expect that they can provide sufficient and varied information about the barriers to and enablers of adopting healthy lifestyle practices, as well as their own lifestyle experiences and possible suggestions for barriers. 
2. For randomized control trial
Bank workers with age ≥18 years old and who fulfil at least one of the following National Cholesterol Education Program Adult Treatment Panel III (NCEP: ATPIII) metabolic syndrome criteria; Waist circumference > 102 cm in men, > 88 cm in women, Triglycerides ≥150 mg/dl, HDL-cholesterol < 40 mg/dl in men and < 50 mg/dl in women, BP ≥130/85 mmHg, fasting glucose ≥110 mg/dl will be included in the study. On the other hand, individuals with dietary restrictions and absolute contraindication for physical activity due to musculoskeletal, neurological, vascular, lung and cardiac problems, pregnant mothers, lactating mothers, those who have a plan to be pregnant within the intervention months and diagnosis of severe psychiatric disorders, significant cognitive impairment, and those who will not available throughout the program will be excluded from the study. Even though they do not meet the criteria for metabolic syndrome and are not included in the study, all individuals who were included for screening and found to be free from any components of metabolic syndrome will receive healthy lifestyle booklets and advise to maintain their lifestyle. 
Bank employees are chosen as participants in this intervention trial because studies done among bank employees in Ethiopia revealed that the prevalence of metabolic syndrome is significant and that rapid intervention is needed for this population. E.g., a cross-sectional study conducted in Ethiopia among 1,935 bank employees and teachers found that 12.5% of participants had metabolic syndrome, and it recommended for more efforts to screen for, identify, and treat metabolic syndrome and its components among Ethiopian bank employees [20].  Cross-sectional research (I am one of the authors) conducted on the prevalence and contributing factors of undiagnosed hypertension among bank employees in Bahir Dar town, Ethiopia, also found that the prevalence of undiagnosed hypertension among 513 bank employees was 24.8%, and that low levels of knowledge about hypertension and physical inactivity were the main risk factors for the development of hypertension (21). This study suggested that creating awareness, frequent screening and apply the right interventions for this vulnerable group is crucial. Another cross-sectional study done among 368 bank employees in Amhara metropolitan cities (Bahir Dar, Gondar, and Dessie) also found that the prevalence of hypertension was 52.4 %, and that variables such as overweight and obesity, daily fruit consumption, moderate to vigorous physical activity, the presence of stressful events, and inadequate cardiovascular diseases knowledge were all linked to hypertension [22]. This study recommended that raising awareness about cardiovascular disorders and behavior change interventions that enhance bank workers engagement in physical exercise, and a healthy diet is urgently required for this group of population.
The other reasons to select bank workers as participants is that they are relatively stable workforce and are likely to be willing to participate in the study. 
Cultural and Social Considerations/Sensitivities  
What cultural and/or social considerations/sensitivities are relevant to the participants in this research project? 
Since, this study will be conducted among educated participants from similar cultures and languages, the intervention will be educational, and the data collection tools do not include any sensitive topics in either qualitative interview guidelines or interventional studies, there won't be any particular cultural and social sensitivities. However, some discomfort may be felt during the data collection period when respondents are asked about their alcohol and smoking habits, due to concerns about social stigma. This will be minimized by assuring them that all information obtained from them will be kept private and secure using a password without their identities having to be recorded. We will also give participants information sheets and let them know that they can leave the study at any time if they feel uncomfortable. In order to lessen the bad feelings associated with failing at the intervention or being unable to adhere to the recommendations for a healthy lifestyle, we will also explain to them that the only negative consequences of failing at the intervention will be to their health. Also, throughout the education sessions, participants will not discuss on their experiences with drinking, smoking, or other bad lifestyle choices.
Incentives  
Will participants be paid or any incentives offered? If so, provide justification and details. The participants will not be paid any incentive for their participation in this study. 
Benefits  
What are the anticipated benefits of the research? I expect that, this study will provide evidence about the barriers and opportunities to implementing healthy lifestyle strategies and the feasibility, acceptability and effect of lifestyle intervention on the metabolic syndrome, the level of knowledge, attitude and practice towards lifestyle and cardiovascular risk factors among office employees with metabolic syndrome in Ethiopia. A tested intervention will also be available for future large-scale implementation trials in the region.
The participants will also have the opportunity to know their metabolic syndrome status and receive lifestyle advice. Additionally, families of the participants will benefit by sharing knowledge and experiences from the participant, and the entire community will also be benefited from the intervention study by preventing them from metabolic syndrome if the intervention is successful in this trial study and utilized as the evidence for development of policy.  
To whom will the benefits flow? 
If the study is successful, policy makers and stakeholders will use it as a planning tool to scale up this management method for metabolic syndrome. Researchers will also use it as a baseline for another large-scale randomized control trial investigation. Additionally, it will be used as evidence for professionals to apply this intervention for risky groups. Moreover, the benefits will also go to the participants by giving the opportunity to know their metabolic syndrome status and receive lifestyle advice, and families of the participants will be benefited from knowledge and experience sharing, the entire community will be benefited from the policy that is developed based on the findings of this randomized control trial. 
Potential Risks  
Indicate if you consider there are any potential risks associated with the proposed procedures, whether to participants or to researchers. Tick the appropriate risk categories.  
Negligible harm No 
Physical harm No 
Psychological harm No 
Devaluation of personal worth No 
Social harm No 
Economic harm No 
Legal harm No 
Other No 
Provide details of the risks associated with the research. 
Some discomfort may be felt during the data collection period when respondents are asked about their alcohol and smoking habits, due to concerns about social stigma. 
To whom do the risks apply? 
The risks may be happened on the participants 
What, if any, strategies will be used to negate, minimize and manage these risks? The risks will be minimized by assuring them that all information obtained from them will be kept private and secure using a password without their identities having to be recorded. We will also give participants information sheets and let them know that they can leave the study at any time if they feel uncomfortable. In order to lessen the bad feelings associated with failing at the intervention or being unable to adhere to the recommendations for a healthy lifestyle, we will also explain to them that the only negative consequences of failing at the intervention will be to their health. Also, throughout the education sessions, participants will not discuss on their experiences with drinking, smoking, or other bad lifestyle choices. 
Justification  
Indicate how the benefits of the research justify the risks. 
This study will provide evidence about the barriers and opportunities to implementing healthy lifestyle strategies and the feasibility, acceptability and effect of lifestyle intervention on the metabolic syndrome, the level of knowledge, attitude and practice towards lifestyle and cardiovascular risk factors among office employees with metabolic syndrome in Ethiopia. 
The participants will also have the opportunity to know their metabolic syndrome status and receive lifestyle advice. Additionally, families of the participants will benefit by sharing knowledge and experiences from the participant, and the entire community will also be benefited from the intervention study by preventing them from metabolic syndrome if the intervention is successful in this trial study and utilized as the evidence for development of policy. 
Informed Consent  
Indicate how informed consent will be obtained from participants. At least one of the following boxes MUST be ticked 'Yes'.  
In writing Yes 
Return of survey or questionnaire No 
Orally No 
Other No 
If Oral Consent or Other, provide details.  
Will consent be obtained on behalf of a participant with impaired capacity? If so, advise from whom consent is being sought. No 
Will consent be obtained from other interested parties? If so, describe why this is to be done and outline the process to obtain this consent. No 
Confidentiality  
For the collection of information, please tick the appropriate categories. At least one of the following boxes MUST be ticked 'Yes'.  
In what form will personal information be collected?  
Identified information No 
Re-identifiable / coded information Yes 
Non-identifiable No 
In what form will personal information be stored?  
Identified information No 
Re-identifiable / coded information Yes 
Non-identifiable No 
In what form will personal information be published or reported?  
Identified information No 
Re-identifiable / coded information No 
Non-identifiable Yes 


Describe the procedures that will be adopted to ensure confidentiality during the collection phase and in the publication of results. 
The confidentiality will be maintained by collecting data without recording the identity of the participants after getting written consent, the data will be stored anonymously with a code number. The only people who will see data are the lead researcher and the supervisors, and identifying details will be stored separately from the rest of the research data. Personal identifying information will be removed from study materials as soon as possible. The identities of the participants or any sensitive information will not be utilized when the study is published. Absolute confidentiality may not be maintained during group education sessions because some participants may work at the same bank branch or may even know one another from the workplace, but group rules will be established prior to the start of group education on maintaining co-workers' secrets. Participants will be clearly informed of this limitation before agreeing to join the study. 
If participants are to be identifiable, or potentially identifiable in any publication or report, outline the procedures for participants to authorize the release of their responses / information and to confirm the accuracy of attributed comments.  
Will a recording of participants be made? No 
If yes, for what purpose will this recording be used?
Will this be retained and used beyond the initial transcription / analysis or will it be erased following transcription?
How will confidentiality be ensured?
How will specific consent for any subsequent use be obtained?  
Data Storage Procedures  
Provide an overview of the data storage procedures for the research. Include security measures and duration of storage. 
The research data will be retained and securely stored for 5 years after publication at the Australian National University. Data will be securely stored on password-protected Australian National University computer at national center for epidemiology and population health. Hard copy documents will be kept in a lockable file cabinet at my office. 


Debriefing  
Will participants be debriefed at the completion of the research? Provide details and include agencies to whom participants may be referred if they have been distressed by the procedures. 
At the conclusion of the data collection and intervention, there will be conversations with the participants, and if there is any distress related to the intervention or the data collection, we will connect them with the Bahir Dar university specialized hospital. However, we can guarantee that the intervention will not cause any anxiety because it is an educational intervention and the data collection will only involve straightforward physical measurements and self-administered questionnaires. 
Feedback  
Provide details of how the results of the research will be reported / disseminated, including the appropriate provision of results to participants. If appropriate, provide details of any planned debriefing of participants. The final result of the study will be submitted to both the Amhara public health institution and the district office of each bank. Each participant will also receive the results through their previously registered information, which will be kept separate from the other research data. The results of the study will also be submitted for publication in scholarly journals. 
Supporting Documentation  
Have you uploaded all relevant supporting documentation, such as Participant Information Sheet and/or consent form, to the documents tab?
 Yes 
Information Sheet  
Consent forms  
Has this work been approved by another Human Research Ethics Committee (HREC)? Yes 
If yes, please give the name of the approving HREC. You will also need to include a copy of the approval letter in your application and also upload an electronic copy to the Documents tab. 
College of Medicine and Health Sciences, Bahir Dar University 
Funding  
Is this research supported by external funding? No 
Provide the name/s of the external sources of funding. Please include grant number/s if available.  
Is the research conducted under the terms of a contract of consultancy agreement between the ANU and the funding source? No 

Describe all the contractual rights of the funding source that relate to the ethical consideration of the research. 
Full Questions Summary 

	Question 	Answer 	
	If this proposal has been reviewed and approved by any other Human Research Ethics Committee, please complete the Expedited Review.
Please read the list carefully and tick 'Yes' to any of the below that relates to your research.	Yes	
	Healthy members of the community	No	
	University students	No	
	Employees or officers of a specific company or organization	Yes	
	Members of a specific community group, club or association	No	
	Clients of a service provider	No	
	Children and young people	No	
	Members of a socially disadvantaged group	No	
	People who belong to a group	No	
	People who may be involved in illegal activities	No	
	People whose primary language is other than English	Yes	
	People in other countries	Yes 	
	Aboriginal and / or Torres Strait Islander Peoples	No	
	People in a dependent or unequal relationship	No	
	Hospital in-patients	No	
	People highly dependent on medical care who may be unable to give consent	No	
	People with a cognitive impairment, an intellectual disability, or a mental illness	No	
	Women who are pregnant and the human fetus	No	
	Bodies or body organs, human tissue or samples	No	
	Other	No	
	Children (under 14)	No	
	Young People (aged 14 - 17)	No	
	Adults (aged 18 or over)	Yes	
	Adults (aged 60+)	Yes	
	Anonymous questionnaires or surveys	Yes	
	Coded (potentially identifiable) questionnaires or surveys	Yes	
	Identifiable questionnaires or surveys	No	
	Observation (overt)	No	
	Observation (covert)	No	
	Interviews (structured or unstructured)	Yes	
	Telephone Interviews	No	
	Focus Groups	No	
	On-line research	No	
	Taping - audio/video	No	
	Photos	No	
	Individually identifiable data	No	
	Re-identifiable data	Yes	
	Non-identifiable data	No	
	Physical examination of participants (e.g. blood pressure, heart and temperature monitoring)	Yes	
	Collection of body tissues or fluid samples	Yes	
	Procedures involving physical experiments (e.g. exercise, reaction to computer images)	Yes	
	Procedures involving administration of substances (e.g. drugs, alcohol, food)	No	
	Examination of medical, educational, personnel or other confidential records	No	
	Surgical procedures	No	
	Clinical Trial	No	
	Comparison or evaluation of clinical procedures	No	
	Comparison or evaluation of counselling or training methods	Yes	
	Comparison of evaluation of drugs or surgical or other therapeutic devices	No	
	Investigation of effects of an agent (drug or other substance)	No	
	Other	No	
	Does this research involve human gametes (eggs or sperm)?	No	
	Does this research involve excess ART embryos?	No	
	Does your proposal involve the collection, use or disclosure of personal information WITHOUT the consent of the participant?	No	
	Does your proposal involve deception of participants, concealment and/or covert observation?	No	
	Does your proposal require the approval of another agency?	No	
	Does the researcher have a conflict of interest, or perceived conflict of interest?	No	
	Are there any other ethical issues associated with the research that you wish to bring to the attention of the HREC?	No	
			
Full Questions Detailed 
Employees or officers of a specific company or organization Yes 
People whose primary language is other than English Yes 
Will participants be provided with written information in the language in which the research will be conducted? Yes 
If not, explain why? 
Indicate in which language(s) the research will be conducted? By Amharic Language 
Will the literacy level of the potential participant pool likely be an issue? No 
If the research is being conducted in a language unfamiliar to either the participant or researcher, what steps will be taken to ensure that participation is given freely and voluntarily? The research will be conducted by language which is familiar for both the researcher and participants.  
Adults (aged 18 or over) Yes 
Adults (aged 60+) Yes 
Anonymous questionnaires or surveys Yes 
Interviews (structured or unstructured) Yes 
Physical examination of participants (e.g. blood pressure, heart and temperature monitoring) Yes 
Collection of body tissues or fluid samples Yes 
Procedures involving physical experiments (e.g. exercise, reaction to computer images) Yes
Examination of medical, educational, personnel or other confidential records? No
  Clinical Trials 
Criteria for Inclusion 
Participants working in both private and government banks in Bahir Dar town and who will be willing to participate and age ≥18 years old and who fulfil at least one of the following National Cholesterol Education Program Adult Treatment Panel III (NCEP: ATPIII) metabolic syndrome criteria; Waist circumference > 102 cm in men, > 88 cm in women, Triglycerides ≥150 mg/dl, HDL-cholesterol < 40 mg/dl in men and < 50 mg/dl in women, BP ≥130/85 mmHg, Fasting glucose ≥110 mg/dl will be included in the study.
Criteria for Exclusion
 Individuals without metabolic syndrome, and those with metabolic syndrome but with dietary restrictions and absolute contraindication for physical activity due to musculoskeletal, neurological, vascular, lung and cardiac problems, pregnant mothers, lactating mothers, who intend to become pregnant during the intervention months and diagnosis of severe psychiatric disorders, significant cognitive impairment, and those who will not available throughout the program will be excluded from the study. 
Has a risk assessment been undertaken by the proposer? No 
If yes, give details of the assessment process No 
Give details of sponsor's insurance No sponsors 
Could this work cause damage to the university's reputation No 
If yes, please give details No 
Does your Clinical Trial involve a drug or device? No 
The trial must be registered with the Australian New Zealand Clinical Trials Registry, has this been done? Yes
If yes, state the name of the Registry: Australian New Zealand Clinical Trials Registry (ANZCTR).

If yes, state the Registration number: ACTRN12623000409673p
If no, state the reasons why trial registration has not been undertaken 
If your clinical trial involves a DRUG OR DEVICE, you will need to provide a copy of your protocol (with attachments) to the Insurance Office. Please contact the Insurance Office on extension 58734 or insurance.office@anu.edu.au for further information. No
Supporting Documentation 
Please ensure electronic copies of the supporting documentation have been uploaded into the documents tab of your protocol. 
These may include (please circle the relevant answer): 
List of indicative questions Y/N =Yes
Copy of questionnaire / survey Y/N=Yes 
Invitation or introductory letter/s Y/N =Yes
Publicity material (posters etc.) Y/N =no 
Information sheet Y/N=Yes 
Consent form Y/N=Yes 
External approval documentation Y/N =Yes
Research visa (if applicable) Y/N=not applicable 
Other (specify below) Y/N= No 
For other, please specify:

SIGNATURES AND UNDERTAKINGS 

PROPOSER OF THE RESEARCH 

I certify that all the persons listed in this protocol have been fully briefed on appropriate procedures and in particular that they have read and are familiar with the national guidelines issued by the National Health and Medical Research Council (the National Statement on Ethical Conduct in Human Research 2007).
 

 
I certify that the above is as accurate a description of my research proposal as possible and that the research will be conducted in accordance with the National Statement on Ethical Conduct in Human Research 2007. I also agree to adhere to the conditions of approval stipulated by the ANU Human Research Ethics Committee (HREC) and will cooperate with HREC monitoring requirements. I agree to notify the Committee in writing immediately of any significant departures from this protocol and will not continue the research if ethical approval is withdrawn and will comply with any special conditions required by the HREC. 


Signed: ................................................. Date: ................... 


ANU SUPERVISOR 

I certify that I shall provide appropriate supervision to the student to ensure that the project is undertaken in accordance with the undertakings above: 
 

Signed:................................................. Date:................... 

 


AS FROM MONDAY 21ST OCTOBER 2013 THE SIGNATURE OF THE HEAD OF ANU DEPARTMENT/GROUP/CENTRE IS NO LONGER REQUIRED. 
