# Supplementary material for: The effectiveness, feasibility, and acceptability of an education intervention promoting healthy lifestyle to reduce risk factors for metabolic syndrome, among office workers in Ethiopia: A protocol for a randomized control trial study
Source: PLoS One. 2024 Aug 30;19(8):e0307659. doi: 10.1371/journal.pone.0307659 (PMC11364252; doi:10.1371/journal.pone.0307659)
Supplement: S5 File — (DOCX) [file pone.0307659.s005.docx]

**Participant Information Sheet for randomized control trial study**

**Researcher:**

My name is **Sitotaw Bogale** and I am a PhD candidate in National Centre for Epidemiology and Population Health at the Australian National University.

**Project Title:** The effectiveness, feasibility, and acceptability of an education intervention promoting healthy lifestyle to reduce risk factors for metabolic syndrome, among office workers in Ethiopia: A randomized control trial.

**General Outline of the Project:**

**Description and Methodology:**

I am conducting research on the effectiveness, feasibility, and acceptability of an education intervention promoting healthy lifestyle to reduce risk factors for metabolic syndrome, among office workers. One of the prevention mechanisms of metabolic syndrome is education regarding the lifestyle, that can bring behavior change. A lifestyle educational intervention is an intervention that help people adopt and maintain behaviors connected to a lifestyle that promotes health by increasing knowledge, self-efficacy, and bringing about behavior change. However, the effectiveness, feasibility and acceptability of educational intervention for healthy lifestyle have not been developed and tested in Ethiopia. As well, knowledge, attitudes and practice towards healthy life style have not been assessed in Ethiopia. Thus, this study aims to assess the effectiveness, feasibility, and acceptability of an education intervention promoting healthy lifestyle to reduce risk factors for metabolic syndrome, among bank workers, as well as the level of knowledge, attitude and practice towards cardiovascular risk factors and healthy lifestyle among bank employees with metabolic syndrome in Ethiopia.

**Participants:** Each group will consist of 113 bank workers. As a result, 226 bank workers will take part in this study.

**Use of Data and Feedback:** The data will be used to produce thesis, peer-reviewed published articles, and conference presentations. The results of the research will be delivered to participants via their previously registered address.

**Participant Involvement:**

**Voluntary Participation & Withdrawal:** Your involvement in this study is voluntary, and you may decline to participate in the study at any time up until the work is ready for publication. You may also withdraw from the study at any time without giving a reason. You can choose not to respond to any of the research questions or giving samples. If you withdraw from the study, the information or sample you supplied before withdrawing will be deleted and not used.

**What does participation in the research entail?**

You are invited to take part in this randomized control trial about the effectiveness, feasibility, and acceptability of an education intervention promoting healthy lifestyle to reduce risk factors for metabolic syndrome, as well as the level of knowledge, attitude and practice towards lifestyle and cardiovascular risk factors. At the start of the study, you will be asked to give a sample of 5 ml blood, have waist circumference measured, and have your blood pressure taken. You will also be asked about your knowledge, attitude, and practice about a healthy lifestyle and cardiovascular risk factors. The total length of intervention period is 9 months and at the ninth month of the intervention, the same exact approach will be used to collect 5ml of blood, measure your blood pressure, waist circumference and ask you questions about your knowledge, attitudes, and practices towards healthy lifestyle a n d cardiovascular risk factor. At the sixth month of the study, you will also be asked questions about the feasibility and acceptability of an educational intervention on lifestyle. You will also receive texts asking you whether you followed the intervention's recommendations’ every two weeks. You will be requested to reply to texts, ask questions, and seek help. Also, you will be invited to attend review sessions, which will be held at the 3^rd^ and 6^th^ months of the intervention and last a half-day or six hours.

**Location and Duration:** Education intervention about leading a healthy lifestyle will last around 1:30 day and be given one-on-one based in your office or any other place you choose. Your waist circumference, blood pressure, and blood sample will be taken at your workplace or another location you choose and will take about 30 minutes. Data on your knowledge, attitude, and practice about a healthy lifestyle and cardiovascular risk factors will be collected at hall on the day of education intervention and will take around 45-minutes. As well as data on the feasibility and acceptability of the intervention, will be gathered during the review meeting that will be held at the 6^th^ month of the intervention, and it will take around 45-minutes.

**Risks**: There will be certain risks associated with uncomfortable questions about lifestyle practices, blood sample draws, physical measures and failure to alter lifestyle and health indicators.

**Benefits**: Through participating in this study, you will receive counselling and education from health experts on healthy lifestyle components which could potentially benefit your health. Your family will also be benefited from your knowledge and experience about healthy lifestyle. It will also help in achieving the objectives of the study, and the community will gain benefit from the policy that will be designed to prevent metabolic syndrome based on the findings of the study.

**Confidentiality:** Because some of you may work at the same bank branch or perhaps know one another from workplace, complete confidentiality may not be upheld. Unless you choose to be named, your identity will not be included in the research. We can use a nickname if you prefer to keep your identity confidential. We will encourage all participants to not discuss the intervention or any individuals involved outside. But we cannot confirm complete confidentiality. Your data will only be accessible to the research team, and identifiable information will be kept apart from the rest of the data. Unless you have chosen differently, results will only be reported in collective and will not identify you specifically.

**Privacy Notice**: In collecting your personal information within this research, the ANU must comply with the Privacy Act 1988. The ANU Privacy Policy is available at <https://policies.anu.edu.au/ppl/document/ANUP_010007> and it contains information about how a person can: Access or seek correction to their personal information; Complain about a breach of an Australian Privacy Principle by ANU, and how ANU will handle the complaint

**Data Storage:**

Data will be securely stored on password-protected computers in the National Centre for Epidemiology and Population health at the Australian National University. Hard copies of records will be kept in a locked filing cabinet in my office. All research data will be kept for at least five years after publications resulting from the study and securely stored. Following the storage term, all personally identifying information will be eliminated from the data, which will then be archived at the Australian Data Archive ([www.ada.edu.au](http://www.ada.edu.au/)) for use in future research, may be by other researchers as well.

**Queries and Concerns:**

Contact Details for More Information: Any requests for information or queries regarding the study participants should be directed to (+251910325473) or my supervisor Dr Matthew Kelly (, +61261250714).

**Ethics Committee Clearance:** The Australian National University Human Ethics Committee and College of Medicine and Health Sciences Bahir Dar University Institutional Review Board approved this study 2022/845 and 792/2023 respectively.
